# Novel routes towards bioplastics from plants: elucidation of the methylperillate biosynthesis pathway from *Salvia dorisiana* trichomes

**DOI:** 10.1093/jxb/eraa086

**Published:** 2020-02-24

**Authors:** Esmer Jongedijk, Sebastian Müller, Aalt D J van Dijk, Elio Schijlen, Antoine Champagne, Marc Boutry, Mark Levisson, Sander van der Krol, Harro Bouwmeester, Jules Beekwilder

**Affiliations:** 1 Wageningen University, Laboratory of Plant Physiology, Wageningen, The Netherlands; 2 Bioinformatics Group, Wageningen University and Research, Wageningen, The Netherlands; 8 Biometris, Wageningen University, Wageningen, The Netherlands; 3 Wageningen Plant Research, 6700 AA, Wageningen, The Netherlands; 4 Louvain Institute of Biomolecular Science and Technology, University of Louvain, Louvain-la-Neuve, Belgium; 5 Arrhenius laboratories, Department of Ecology, Environment and Plant Sciences, Stockholm University, Stockholm, Sweden; 6 Plant Hormone Biology Group, Swammerdam Institute for Life Sciences, University of Amsterdam (UVA), Amsterdam, The Netherlands; 7 Michigan State University, USA

**Keywords:** Biobased commodity chemicals, glandular trichome, limonene-7-hydroxylase, methyl carboxyl ester, methylperillate, methyltransferase, monoterpene biosynthesis, *Nicotiana benthamiana*, *Salvia*

## Abstract

Plants produce a large variety of highly functionalized terpenoids. Functional groups such as partially unsaturated rings and carboxyl groups provide handles to use these compounds as feedstock for biobased commodity chemicals. For instance, methylperillate, a monoterpenoid found in *Salvia dorisiana*, may be used for this purpose, as it carries both an unsaturated ring and a methylated carboxyl group. The biosynthetic pathway of methylperillate in plants is still unclear. In this work, we identified glandular trichomes from *S. dorisiana* as the location of biosynthesis and storage of methylperillate. mRNA from purified trichomes was used to identify four genes that can encode the pathway from geranyl diphosphate towards methylperillate. This pathway includes a (–)-limonene synthase (SdLS), a limonene 7-hydroxylase (SdL7H, CYP71A76), and a perillyl alcohol dehydrogenase (SdPOHDH). We also identified a terpene acid methyltransferase, perillic acid *O-*methyltransferase (SdPAOMT), with homology to salicylic acid OMTs. Transient expression in *Nicotiana benthamiana* of these four genes, in combination with a geranyl diphosphate synthase to boost precursor formation, resulted in production of methylperillate. This demonstrates the potential of these enzymes for metabolic engineering of a feedstock for biobased commodity chemicals.

## Teaser

Monoterpene methylperillate can be used efficiently as a convenient starting material for polyethylene terephthalate plastic. We describe a biosynthetic pathway towards methylperillate from *Salvia* and report enzymes for key reactions.

## Introduction

Plants are a rich source of workable materials such as wood, rubber, and cotton. Since the second half of the 19th century, many plant-based materials have been replaced by synthetic alternatives, derived from fossil carbon, often with new and superior properties. Currently, concerns about the exploitation of fossil resources are growing (Cho *et al.*, 2020). Therefore, new opportunities arise for biobased materials which can be used as sustainable substitutes for petrochemical building blocks.

Recently, it was suggested that plant monoterpenoids can be deployed for creating commodity chemical building blocks (Colonna *et al.*, 2011; Jongedijk *et al.*, 2016). For example, the monoterpenoid methylperillate (MePA) can be converted to the commodity chemical terephthalic acid (TA) in only two steps (Jongedijk *et al.*, 2018). The main application of TA is as building block for polyethylene terephthalate (PET) plastics, and its consumption is expected to reach 65 Mt in 2018 (Collias *et al.*, 2014). TA is currently produced from oil, through oxidation of para-xylene (Rezaei and Sajadi, 2015).

Plant species from the Lamiacae family are rich in monoterpenoids, often derived from limonene. MePA is naturally present in the plant species *Salvia dorisiana* (Halim and Collins, 1975; Conti *et al.*, 2012), whose essential oil contains up to 20% MePA, and the oil yield is 1–2 g oil 100 g^–1^ DW biomass (Jongedijk *et al.*, 2018). Although production of MePA could be of interest as a precursor for biobased materials, *S. dorisiana* is not yet suited to be adopted as an agricultural crop. A sustainable (green) production process in heterologous hosts such as microorganisms or other plant species could be envisaged, but the biosynthetic pathway to MePA has still not been fully elucidated.

**Fig. 1.**
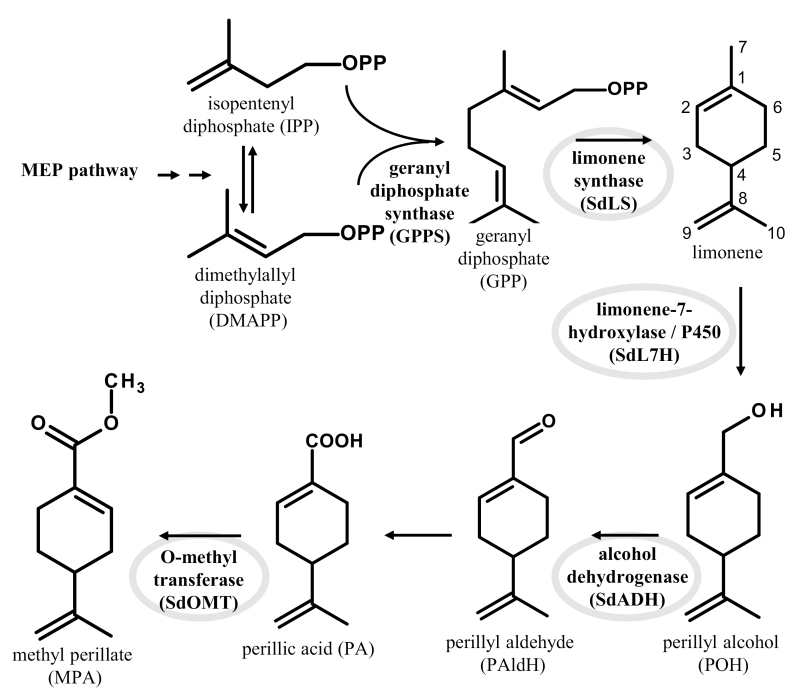
Methyl perillate biosynthetic pathway in *Salvia dorisiana* trichomes as identified in this study. Enzymatic reactions range from limonene biosynthesis, limonene hydroxylation on the C7-position, subsequent oxidations, and finally carboxylic acid methylation. Conversion of perillyl alcohol to perillic acid was mediated by endogenous *N. benthamiana* activity.

Monoterpenoids are C10 compounds, derived from geranyl diphosphate (GPP) that is produced through either the plastidic methylerythritol phosphate (MEP) pathway or the cytosolic mevalonate pathway. MePA is a monoterpenoid with a structure related to the cyclic monoterpene limonene, but carrying a methylated carboxyl group (Fig. 1). Biosynthesis of monoterpene alcohols and aldehydes has been extensively studied, for example of menthol and carvone (Lange, 2015). The biosynthesis of carboxyl groups has mainly been studied for sesquiterpenes and diterpenes, such as artemisinic acid (Teoh *et al.*, 2006), germacrene A acid (de Kraker *et al.*, 2001; Nguyen *et al.*, 2010; Cankar *et al.*, 2011), and abietic acid (Ro *et al.*, 2005). Also the formation of carboxyl groups in the pathway towards the indole alkaloid strictosidine has been characterized (Miettinen *et al.*, 2014). Formation of carboxyl groups in monoterpenoids is much less known, except for chrysanthemic acid formation in pyrethrum (*Tanacetum cinerariifolium*), which was recently elucidated (Ramirez *et al.*, 2012; Xu *et al.*, 2018). Biosynthesis of methyl esters has been reported for monoterpene indole alkaloids (Murata *et al.*, 2008), but these molecules hardly compare with perillic acid (PA)-like monoterpenes. Therefore, elucidating the MePA pathway cannot easily be inferred by homology to related pathways in other species.

For an efficient identification of the MePA pathway, it is essential to understand its localization in the plant. Biosynthesis of monoterpenes usually takes place in specialized tissues or cells (Lange and Turner, 2013). In Lamiacae species, monoterpene olefins, alcohols, and ketones are generally produced in specialized glandular trichomes on the leaf surface (Gershenzon *et al.*, 1992; Iijima *et al.*, 2004; Bumblauskiené *et al.*, 2009). Monoterpene acids are often synthesized in trichomes. In Pyrethrum, the monoterpene chrysanthemol is converted to chrysanthemic acid in the trichomes (Ramirez *et al.*, 2012). In *Artemisia annua*, artemisinic acid is stored in glandular trichomes, together with other terpenoids (Bertea *et al.*, 2005). In addition, the formation of strictosidine in *Catharanthus roseus* is known to involve several cell types, including internal phloem-associated parenchyma, where acid formation takes place, and epidermal cells, where monoterpene acid methylation takes place (Murata *et al.*, 2008; Miettinen *et al.*, 2014).

In this study, we investigated the biosynthesis of MePA in *S. dorisiana* and showed that it occurs in a specific glandular trichome type. Transcript profiling of these trichomes resulted in the identification and characterization of genes involved in MePA formation. Therefore, we elucidated a route of cyclic monoterpene biosynthesis in plants including acid formation and subsequent methylation in the trichomes. Heterologous production of MePA by transient expression of the entire pathway in *Nicotiana benthamiana* was demonstrated, as the first step towards bioplastic precursor production.

## Materials and methods

### Plant material and growth

*Salvia dorisiana* plants were grown in the greenhouse under a 16 h photoperiod, 19–21 °C, and 60% humidity. Plants were multiplied by cutting.

### Trichome isolation

Trichomes of apical buds and young leaves up to 7 cm were isolated as previously described (Gershenzon *et al.*, 1992), with the following adaptations. XAD-4 (Amberlite®, Acros Organics) and 0.5 mm glass beads (unwashed, 425–600 µm, Sigma) were washed on a Büchner funnel with a pump before use, and subsequently with 95% ethanol, 3 M HCl, and demineralized water, three times each. A 15 g aliquot of buds and leaves was soaked for 1 h in ice-cold demineralized water. A bead-beater (Biospec Products, Model 1107900) was filled in with 15–20 g of plant material, 100–130 g of glass beads, XAD-4 beads (1 g g^–1^ plant material), and isolation buffer to full volume as described (Gershenzon *et al.*, 1992); and with a rheostat (Powerstat® variable autotransformer) three pulses of 1 min each of 110 V were given, while continuously cooling the bead-beater with the ice compartment. The content was passed through a 280 μm nylon mesh (Kabel-Zaandam) and washed twice with washing buffer (isolation buffer without methyl cellulose and PVP). The 280 μm filtrate was passed through a 90 μm mesh and the trichome heads were collected by passing the 90 μm filtrate through a 20 μm mesh. The trichome heads were washed in the washing buffer and scraped from the mesh. For subsequent RNA extraction or metabolite analysis, the trichome heads were snap-frozen immediately in liquid N_2_ and kept at –80 °C up to 1 month. For microscopy, the trichome heads were suspended in a small volume of washing buffer and kept at 4 °C for up to 2 d.

### Light microscopy

Photographs of the leaf surface were taken under a Zeiss stemi SV11 binocular, equipped with a Nikon DS-Fi1 digital sight camera. Pictures of isolated trichomes were taken with an inverted microscope (Zeiss Axioskop), at ×10–100 magnification, equipped with the same camera.

### SEM

Small pieces of *S. dorisiana* leaves, including abaxial and adaxial sides, were attached to a brass Leica sample holder with carbon glue (Leit-C, Neubauer Chemikalien, Germany). The holder was affixed on the cryo-sample loading system (VCT 100, Leica, Vienna, Austria) and simultaneously frozen in liquid nitrogen. The frozen holder was transferred to the cryo-preparation system (MED 020/VCT 100, Leica) onto the sample stage at –92 °C. For removal of frost contamination on the sample surface, the samples were freeze-dried for 5 min at –92 °C and 1.3×10^−6^ mbar. After sputter coating with a layer of 20 nm tungsten at the same temperature, the sample holder was transferred to the field emission scanning electron microscope (Magellan 400, FEI, Eindhoven, The Netherlands) onto the sample stage at –120 °C. The analysis was performed with SEM detection at 2 kV and 6.3 pA. SEM pictures were taken at a magnification of ×200–3500 (scale bars are indicated in the respective images).

### Metabolite quantification

*Salvia dorisiana* plant tissues were harvested in three biological replicates. The FW/DW ratio was determined by drying at 105 °C for 20 h. To measure metabolites, the plant tissues were frozen in liquid N_2_, ground in a mortar and pestle while still frozen, and 1 ml of ethyl acetate, with 3 µg ml^–1^ γ-terpinen-4-ol as internal standard, was added to 100 mg of frozen plant powder, vortexed for 20 s, subjected to sonication for 5 min, and centrifuged for 5 min at 3400 rpm. The supernatant was analysed by GC-MS as described below. A standard mix of 3 µg ml^–1^ terpinen-4-ol, (*R*)-(+)-limonene (97%, Sigma Aldrich), perillyl alcohol (96%, Sigma Aldrich), (*S*)-(–)-perillaldehyde (Sigma Aldrich), and (*S*)-(–)-perillic acid (95%, Sigma Aldrich) was prepared for identification and semi-quantification. The standard mix was methylated by addition of diazomethane 1:1 and injected as well, to be able to identify and semi-quantify MePA in the samples.

### Extracting trichomes by chloroform dipping

Chloroform dipping was performed according to Ramirez *et al.* (2012). A concentration of 3 µg ml^–1^ (–)-terpinen-4-ol (Aldrich) was added to the chloroform as an internal standard. Four fresh leaves <3 cm were weighed and submerged in a time series in glass tubes, in 10 ml of CHCl_3_ for 5 s, another 10 ml for 10 s, and another 10 ml for 15 s. Leaves were immediately snap-frozen in liquid N_2_ after dipping. Intact leaves and leaves after dipping were extracted with chloroform and dried, as described under ‘GC-MS of extracts’. GC-MS peak areas of the time series were put together. Fresh weight was determined by weighing the whole leaf in the case of the content of the chloroform dip (D), and weighing the amount of ground leaf powder in liquid N_2_ in the case of intact leaves (L) and leaves after dipping (DL).

### RNA extraction, cDNA preparation, and cDNA sequencing

Plant tissues were ground with a pestle and mortar while still frozen in N_2_, and transferred to a pre-cooled tube in liquid N_2_. RNA was isolated with the RNeasy Plant Mini kit (Qiagen) according to the manufacturer’s protocol. For sequencing, RNA was treated with DNase I (Invitrogen). cDNA for cloning was prepared with the SMART RACE cDNA amplification kit (Clontech, Palo Alto, CA, USA).

### PacBio Iso-Seq sequencing

Preparation of a PacBio Iso-Seq library was performed according to ‘Procedure & Checklist—Isoform Sequencing (Iso-Seq™, Pacific Bioscience) using the Clontech SMARTer PCR cDNA Synthesis Kit and Manual Agarose-gel Size Selection’. In brief, 1 µg of total RNA was used for cDNA synthesis using a SMARTer PCR cDNA Synthesis Kit (Clontech) and diluted with PacBio elution buffer to 90 μl final volume. After optimization, a large-scale cDNA amplification was done using 80 μl of diluted cDNA, 5'PCR primer II A (Clontech), 1 U of Kapa HiFi enzyme, KAPA dNTP, mix, and buffer, with 18 PCR cycles in eight parallel reactions of 50 μl each. Amplified cDNA was pooled and purified using Ampure PB beads. Three aliquots of 500 ng of amplified cDNA were used for size selection on a 0.8% agarose 1× TAE gel. Three different size fractions (0.2–1, 1–2, and 2–4 kb) were purified using a MinElute Gel Extraction Kit (Qiagen). PCR amplification was performed on each isolated fraction as above using 45, 60, and 120 s of elongation and 10, 12, and 15 cycles for the short, medium, and large fraction, respectively. DNA template prep kit v3.0 was used for SMRT bell construction, DNA damage repair, end repair, and adaptor ligation. SMRT bells were further used for primer and polymerase (P5) binding according to the manufacturer’s guidelines (PacBio). Sequencing was done on a PacBio RS-II system with Chemistry v3, one cell per well and stage start. PacBio Iso-Seq analysis was performed using the PacBio SMRT portal software using standard settings.

Filtered PacBio reads were then assembled into clusters using Cap3 (Huang and Madan, 1999). Getorf (Rice *et al.*, 2000) was applied to the resulting clusters (including the remaining singlets) in order to find regions between stop codons with a minimum size of 600 nt. The resulting predicted ORFs were clustered using DNACLUST (Ghodsi *et al.*, 2011) using a value of 0.85 for the similarity threshold between the cluster centre and a given sequence in the cluster, and the option allow-left-gaps. In each of the resulting clusters, MUSCLE (Edgar, 2004) was used for sequence alignment, and a consensus sequence was obtained using the most often occurring nucleotide at each position.

### HiSeq sequencing

Total RNA of plant tissues (leaves <3 cm, leaves 3–10 cm, leaves >10 cm, stem, roots, and trichomes) was prepared. Of each isolation, 1 μg of total RNA was used for mRNA isolation, and subsequent RNAseq library preparation following the TruSeq Stranded mRNA Sample Preparation Protocol (Illumina). In brief, mRNA was isolated using oligo(dT) beads and chemically fragmented prior to first-strand cDNA synthesis using random primers. Strand specificity was achieved by replacing dTTP with dUTP during second-strand synthesis and the addition of actinomycin D to the first-strand master mix. The double-stranded cDNA fragments obtained were used for 3' adenylation and adaptor ligation using different barcoded adaptors. Adaptor-ligated cDNA was finally amplified using 15 PCR cycles. Quality control of final libraries was done using an Agilent Tapestation High Sensitivity D1000 Screen Tape assay. Final library quantification was performed using Qubit (Invitrogen). Equimolar amounts of libraries were pooled and diluted towards 6 pM concentrations for paired end clustering on one lane of a flow cell V4 (Illumina). Final sequencing was performed on a HiSeq2500 instrument using a 126–7–126 flow cycle pattern. De-multiplexing of obtained sequences was done using CASAVA 1.8.1. software.

Reads were mapped with CLC Genomics Workbench 8.0 (Qiagen), using the following settings: one reference sequence per transcript; mismatch cost=2; insertion cost=3; deletion cost=3; length fraction=0.9; similarity fraction=0.9; global alignment=no; strand specific=both; maximum number of hits for a read=10; count paired reads as two=no; expression value=RPKM (reads per kilobase per million mapped reads).

### Identification of candidate genes

The R function cor was used to calculate Pearson correlation between expression levels for each of the sequences resulting from the PacBio and HiSeq sequencing with the *SdLS* gene (that had first been characterized), as well as with the metabolite level in each of the tissues. For the latter, the average level of the different metabolites was used. Pearson correlation values and expression values were visualized using plot3D (Soetaert, 2013).

PFAM domains (Finn *et al.*, 2016) were predicted in each of the sequences resulting from the PacBio sequencing using HMM search (Eddy, 2011). To assess the predictive value of filtering on the two correlations mentioned above and on the expression value, sequences were filtered to have a Pearson correlation of at least 0.6 with *SdLS* and with the metabolite level, and an expression level of at least 1000. Enrichment of PFAM domains in the set of sequences selected in this way compared with the rest of the sequences was assessed using the Fisher exact test as implemented in fisher.test (R) followed by multiple testing correction using the Benjamin–Hochberg procedure. A false discovery rate (FDR) cut-off of 0.05 was applied.

To identify candidate genes for enzymes in the pathway towards MePA, the highly expressed genes were analysed by additional blast analysis in GenNank (Benson *et al.*, 2013). P450 proteins were systematically named by David Nelson (Nelson, 2009).

### Cloning and PCR

Fragments were amplified with Phusion® High-Fidelity DNA Polymerase (NEB) and primers (Supplementary Table S1 at *JXB* online), according to the manufacturer’s protocol. All constructs were first introduced into *Escherichia coli* DH5α, isolated with miniprep, sequenced by Macrogen Europe Amsterdam, and subsequently introduced into their destination strains.

Monoterpene synthase- and methyltransferase-coding sequences were cloned in the pCDF-duet vector (Novagen) for *in vitro* assay as described before (Supplementary Table S7) (Jongedijk *et al.*, 2015). Monoterpene synthases were expressed with the plastid targeting signal removed; methyltransferase sequences were expressed as the full length. When necessary, sequences were amplified to full length by RACE-PCR, according to the manufacturer’s primer design directions and protocol.

The *SdL7H* sequence was cloned in a constructed pTEF plasmid (Supplementary Table S7) for characterization in yeast. *SdL7H* was amplified using primers RT3/RT4 (Supplementary Table S1). Plasmid backbone fragments, containing the URA3 marker, 2µ ori, and the *E. coli* AmpR marker and ori, were amplified using primers RT13/RT2, RT5/RT6, RT7/RT8, and RT9/RT14 (Supplementary Table S1). Expression of *SdL7H* is controlled by the ScTEF1 promoter and terminator. All fragments contained 60 bp homologous sequences and were assembled *in vivo* into the episomal expression vector pTEF1p-SdL7H-Tef1t using transformation-assisted recombination. Correct assembly of the plasmid was confirmed via PCR analysis and sequencing (Macrogen).

Monoterpene synthases, methyltransferases, and GPP synthase (GPPS) sequences were cloned for agro-infiltration in the entry vector ImpactVector pIV1A-1.1 (Supplementary Table S7) (www.impactvector.com), modified with *Not*I/*Pac*I restriction sites at the polylinker, and the original *Pac*I site and existing ATG codon removed (ImpacTim). The construct was transferred by LR reaction to the pBIN+ binary destination vector (Vanengelen *et al*., 1995) according to the manufacturer’s protocol. pBIN+ constructs were used for *Agrobacterium tumefaciens* transformations.

All other sequences were cloned for agro-infiltration in the directional entry vector pENTR™/D-TOPO® (Invitrogen) (Supplementary Table S7) according to the manufacturer’s protocol. Constructs were transferred to pB7WG2 binary destination vector (Karimi *et al.*, 2002) by LR reaction and used for *A. tumefaciens* transformation.

### *In vitro* assays

*In vitro* enzyme expression was performed as described previously (Jongedijk *et al.*, 2015). Monoterpene synthase assay conditions were as described in that study. For the methyltransferase assay, cell supernatants were stored at –20 °C (700 µl of supernatant with 300 µl of 50% glycerol). The assay was performed by mixing 70 µl of cell supernatant/glycerol mix with 50 µl of 500 mM Tris–HCl pH 7.0, 20 µl of 32 mM *S*-adenosyl-L-methionine (SAM), 10 µl of 50 mM substrate [salicylic acid (SA; Acros Organics), PA (95%, Sigma Aldrich), benzoic acid (BA; 99.5%, Sigma Aldrich), or jasmonic acid (JA; >95%, OlChemIm) in DMSO], filled up to 500 µl with MQ. The mix was incubated for 1 h at room temperature with gentle agitation (20 rpm). Tubes were extracted with 2 ml of ethylacetate by vortexing for 20 s and sonicating for 5 min, centrifuged for 5 min (3400 rpm), and the ethylacetate phase was collected, dried over anhydrous Na_2_SO_4_, and analysed by GC-MS. Reference compounds [methyl jasmonate (>95%, OlChemIm), methyl salicylate (99%, Aldrich), PA, and BA] were dissolved in methanol, diluted in ethylacetate to the range of 3–12 µg ml^–1^, and for PA and BA methylated by 1:1 addition of diazomethane.

### Yeast assays

A limonene-producing yeast strain, Sc-PftLS (Jongedijk *et al.*, 2015), was transformed with the pTEF1p-SdL7H-Tef1t vector using the LiOAc/PEG method (Gietz *et al.*, 1995), resulting in strain Sc-PftLS-SdL7H. A single colony was grown as pre-culture at 30 °C and 250 rpm in 10 ml of synthetic medium (6.7 g l^–1^ yeast nitrogen base without amino acids) with 20 g l^–1^ glucose and appropriate growth factors to supplement the specific auxotrophic requirements of the strain. Shake flask cultures, inoculated at an OD_600_=0.4, were grown in an orbital shaker at 250 rpm and at 30 °C in 250 ml flasks containing 50 ml of synthetic medium with 20 g l^–1^ galactose and appropriate growth factors. After 96 h of growth, cultures were analysed for perillyl alcohol (POH) production.

### Alignment (Clustal-W)

Protein sequence alignments were performed with CLC Genomics Workbench version 8.0.2 (Qiagen Aarhus) software using the alignment algorithm with standard parameters: gap open cost=10.0; gap extension cost=1.0; end gap cost=as any other; alignment mode=very accurate (slow); redo alignments=no; use fixpoints=no.

### Transient gene expression in *N. benthamiana*

Constructs for agro-infiltration were transferred to *A. tumefaciens* strain Agl0 by electroporation. Agro-infiltrations were performed as described (Cankar *et al.*, 2015) with the following modifications: medium was supplemented with 50 µg ml^–1^ kanamycin and 40 µg ml^–1^ rifampicillin for pBIN+-based constructs, and with 100 µg ml^–1^ spectinomycin and 40 µg ml^–1^ rifampicillin for pB7WG2-based constructs.

### Compound infiltrations in *N. benthamiana*

Solutions of 400 µM of compounds [limonene, POH, perillyl aldehyde (PAldH), and PA] were prepared in 0.2% DMSO. Compounds were infiltrated on the fifth day after infiltration of *A. tumefaciens* strains, and leaves were harvested 4 h after compound co-infiltration.

### Headspace trapping

Trapping of headspace volatiles was performed as described (Cankar *et al.*, 2015) with the following modifications: headspace sampling was performed in a climate room (20±2 °C, 56% relative humidity) with LED lighting (adjusted at 100% white, 10% deep red, 100% far red, and 5% blue light). Volatiles were trapped by sucking air out of the jar at a rate of 100 ml min^−1^ (inlet flow at 150 ml min^−1^) for 4 h.

Trapped headspace volatiles were analysed using a Thermo TraceGC Ultra connected to a Thermo TraceDSQ quadrupole mass spectrometer (Thermo Fisher Scientific, Waltham, MA, USA). Settings were as described (Jongedijk *et al.*, 2015), except that volatiles were injected on the analytical column at a split ratio of 10.

### GC-MS of extracts

Aliquots of 200 mg of frozen, powdered leaf material were extracted with 2 ml of ethylacetate. Samples were vortexed for 20 s, centrifuged for 5 min at 3400 rpm, and the ethylacetate phase was dried over anhydrous Na_2_SO_4_. GC-MS of extracts was performed on a 7890A gas chromatograph (Agilent) equipped with a mass selective detector (Model 5975C, Agilent) as described previously (Jongedijk *et al.*, 2015).

Yeast cultures were extracted using 5 ml of ethyl acetate by vigorous shaking for 2 min and separation of ethyl acetate in a separation funnel, followed by centrifugation of the ethyl acetate phase (5 min, 3500 *g*) to further clarify the ethyl acetate phase. Extracts were dried over anhydrous Na_2_SO_4_ and analysed by GC-MS, as for plant extracts.

For identification of the enantiomer of limonene, the gas chromatograph was equipped with an enantioselective column with stationary phase Heptakis (6-O-TBMDS-2,3-di-*O*-methyl)-β-cyclodextrin; 50% w/w in OV1701) of 25 m and internal diameter 0.25 mm, with settings as described previously (Jongedijk *et al.*, 2015).

### LC-MS

Aliquots of 200 mg of frozen, powdered leaf material were extracted with 0.6 ml of 99.9% MeOH/0.125% formic acid. After 20 s vortexing and 5 min sonication, the extracts were centrifuged for 20 min at maximum speed in a table-top centrifuge. LC-MS was performed on an Accela HPLC tower connected to a LTQ/Orbitrap hybrid mass spectrometer (Thermo Fisher Scientific), with conditions and settings as described previously (van der Hooft *et al.*, 2012).

### GenBank accession numbers

*SdLS*, MH051312; *SdPS*, MH051313; *SdLiS*, MH051314; *SdPCS*, MH051315; *SdSaS*, MH051316; *SdEuS*, MH051317; *SdL7H_CYP71A76*, MH051318; *SdCYP76AH36*, MH051320; *SdCYP76AK17*, MH051321; *SdCYP71A77*, MH051322; *SdCYP76AK18*, MH051323; *SdPOHDH*, MH051324; *SdPAOMT*, MH051325; *SdOMT3*, MH051328; *SdOMT4*, MH051327; *SdOMT9*, MH051326; *SdOMT18*, MH051329; *SdOMT19*, MH051330. RNA sequencing reads were submitted to NCBI-SRA under number PRJNA595496.

## Results

### Methylperillate and its precursors localize to *S. dorisiana* glandular trichomes

**Fig. 2.**
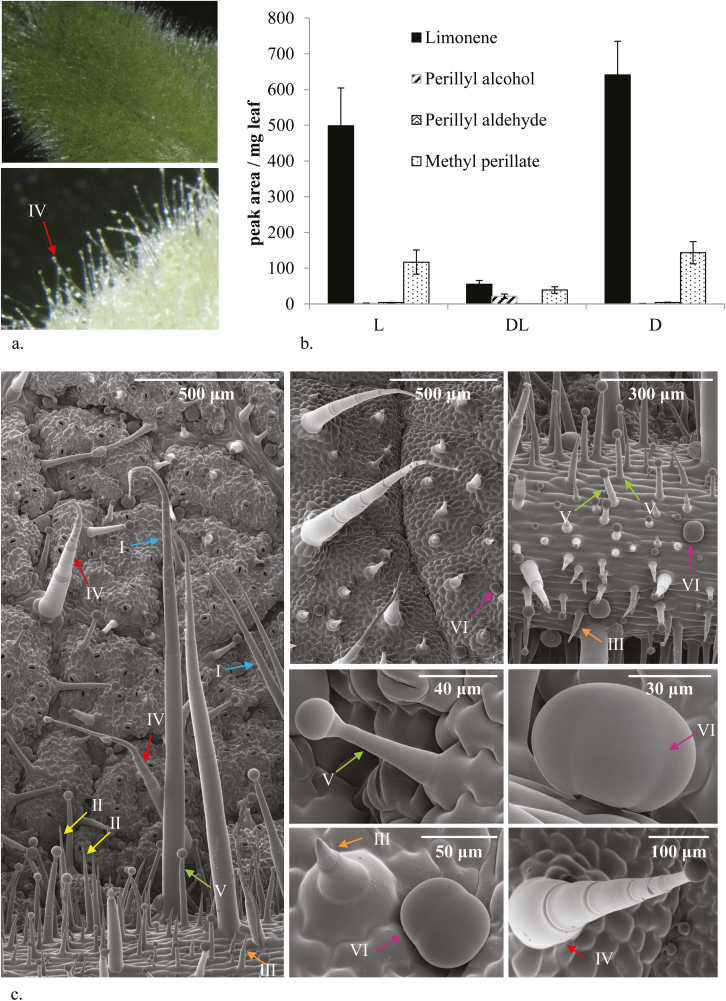
*Salvia dorisiana* trichomes. (a) Light microscopy of *S. dorisiana* leaf surface which is covered in trichomes. (b) Methylperillate pathway compounds are enriched in the trichomes. L, intact leaves; DL, leaves after dipping; D, chloroform dip. Error bars indicate the SD, *n*=3. (c) Cryo-SEM pictures of leaf surface. Arrows indicate different trichome types (Glas *et al.*, 2012): trichomes labelled I, long stalk non-glandular (2 mm);II, intermediate stalk non-glandular (0.5 mm); III, short none-glandular stalk (0.05–0.4 mm); IV, long glandular stalk; V, intermediate glandular stalk; VI, peltate glandular stalk (glandular part very close to leaf surface).

MePA and its precursors limonene, POH, and PAldH (Fig. 1) were analysed in leaves of different developmental stages, stems, and roots of *S. dorisiana* by GC-MS analysis of ethyl acetate extracts of homogenized tissues. Both MePA and the other metabolites were over-represented in small leaves, compared with larger leaves as well as stem and root tissues (Supplementary Fig. S1). Limonene was exclusively present as the (–)-enantiomer, as shown by GC-MS analysis on an enantioselective column (Supplementary Fig. S2). Leaves of *S. dorisiana* are covered with trichomes (Fig. 2a). A chloroform dip extraction on intact leaves was used to extract all chloroform-soluble contents from the trichomes and leaf surface without extracting the leaf interior. Analysis of the chloroform extract showed that MePA and its precursor compounds are mostly present on the surface of the leaf (Fig. 2b).

Trichomes on *S. dorisiana* leaves display several morphologies (Fig. 2c). Six trichome types could be observed in an SEM study, including three non-glandular trichome types: long (arrows labelled with I in Fig. 2), middle-sized (arrows II), and short trichomes (arrows III). Also three types of glandular trichomes were observed: long-stalked capitate trichomes (arrows IV in Fig 2c), short-stalked capitate trichomes (arrows V), and peltate trichomes (arrows VI).

The trichome types of *S. dorisiana* compare well with trichomes of other *Salvia* species (Eiji and Salmaki, 2016). Interestingly, the peltate glandular trichomes have a very short stalk, compared with glandular trichomes from tomato (Glas *et al.*, 2012). For comparison, the glandular trichomes of *Mentha* species also have a very short stalk, but the glandular head consists of eight cells (Turner *et al.*, 2000), instead of four cells which we observe in *S. dorisiana*.

Trichomes from *S. dorisiana* were isolated from fresh leaves (Supplementary Fig. S3a) and analysed by GC-MS, and MePA and its biosynthetic precursors were detected (Supplementary Fig. S3b). *Salvia dorisiana* trichome types were separated by using a combination of meshes, Percoll, and sucrose gradient centrifugation (Sallets *et al.*, 2014), which resulted in a 100 µm ‘hairy fraction’ and a fraction of near-pure peltate glandular trichome heads. Metabolite analysis of the separated trichome fractions showed that the monoterpenes limonene and MePA are most abundant in the peltate glandular trichomes (Supplementary Fig. S4).

### Comparative expression analysis reveals genes involved in *S. dorisiana* trichome metabolism

Subsequently, a study into the genes encoding the metabolic pathway towards MePA was initiated. The observation that MePA and its intermediates localize predominantly to the trichomes suggested that the biosynthetic genes are also specifically expressed in the trichomes. While a cDNA analysis of specific peltate trichomes would be preferred, RNA yields from these issues were generally low and of poor quality. Therefore, cDNA from a total trichome fraction was generated, sequenced by PacBio IsoSeq, and transcripts were assembled. In order to select the genes that are predominantly expressed in trichomes, mRNA expression profiling of different tissues was used and compared with metabolic profiling of the same tissues. For this purpose, short-read HiSeq RNA sequencing was performed on cDNA from trichomes, leaves <3 cm, leaves 3–10 cm, leaves >10 cm, and stems and roots (Supplementary Table S2). Reads were mapped on the previously assembled transcripts, to determine expression levels of the different genes in the different tissue samples.

The collection of cDNA sequences from *S. dorisiana* trichomes was screened for the absolute expression level of each transcript in trichomes, and for correlation of its expression profile over the different tissues with the metabolites profile over the different tissues (as in Supplementary Fig. S1; Supplementary Table S3). To identify candidate genes for enzymes in the pathway towards MePA, PFAM domains and blast results in GenBank were analysed (Supplementary Table S3). First, six candidate monoterpene synthases were extracted from this data set, and cloned for characterization. Six monoterpene synthase candidates were expressed in *E. coli* and characterized by *in vitro* assays, including a β-pinene synthase SdPS, a linalool synthase SdLiS, an α-pinene/camphene synthase SdPCS, a sabinene synthase SdSaS, and a eucalyptol synthase SdEuS (Supplementary Figs S5, S7). Also a limonene synthase, SdLS, was identified, and was shown to produce limonene into the leaf headspace upon agro-infiltration in *N. benthamiana* (Supplementary Fig. S6). Headspace analysis was used here since *N. benthamiana* will not store limonene in its leaves, but will rather emit it into the leaf headspace.

**Fig. 3.**
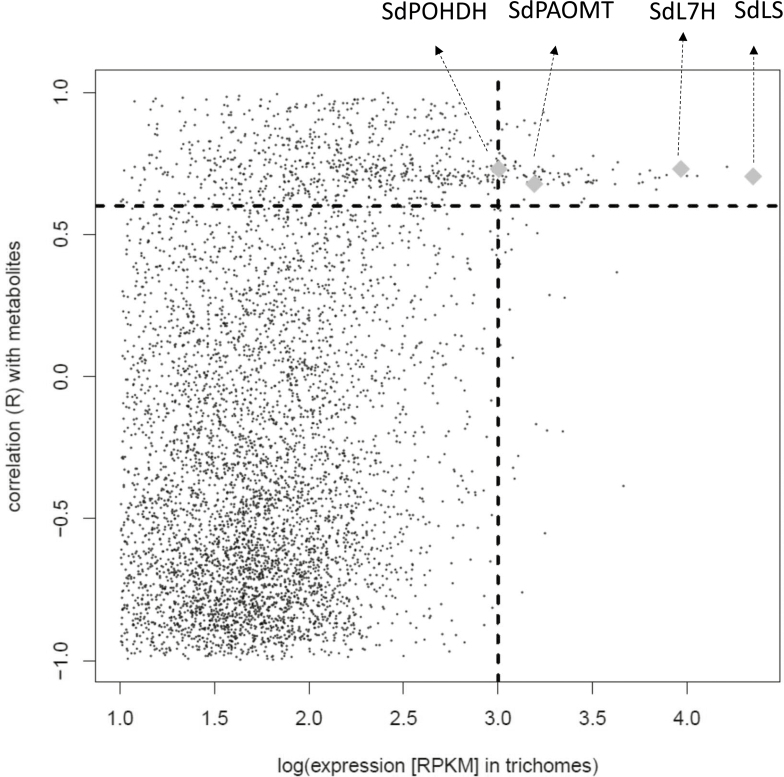
Plot of gene expression patterns in *S. dorisiana* tissues. Expression in trichomes versus correlation with limonene synthase expression pattern versus correlation with metabolite presence. Each dot represents a gene candidate. Interesting gene candidates, with high co-expression with the limonene synthase (*R*>0.6), high correlation with the presence of the pathway metabolites in the tissue (*R*>0.6), and a high absolute expression in the trichomes (RPKM >1000), were carefully considered (black dots). Characterized genes that were functional in methylperillate biosynthesis are indicated (diamonds). SdLS, limonene synthase; SdL7H, limonene-7-hydroxylase (P450); SdPAOMT, perillic acid *O*-methyltransferase; SdPOHDH, perillyl alcohol dehydrogenase.

For identification of downstream genes of the pathway, an additional sorting of transcripts was performed on co-expression with the LS. *SdLS* was the most highly expressed gene in trichomes (Fig. 3). A set of 86 transcripts was selected for their high absolute expression level in the trichomes (using an arbitrary criterion RPKM >1000), high correlation with the presence of pathway metabolites (average weight per FW of pathway metabolites, *R*>0.6), and high co-expression with LS (*R*>0.6) (Fig. 3; Supplementary Table S3; Supplementary dataset S1).

The set of transcripts was sorted on expression level in the trichomes, in order to select candidate genes for P450, alcohol dehydrogenase, and methyltransferase activities (Fig. 1; Supplementary Table S3). The selected set was also analysed for enriched PFAM classes in a non-targeted way. These classes include putative terpene synthases, P450s, methyltransferases, dehydrogenases, acetyltransferases, transporters, ATP synthase, NAD(P)H binding, and some enzyme domains related to fatty acid metabolism (Supplementary Table S4).

### Metabolite markers indicate the formation of pathway intermediates in *N. benthamiana*

The activity of candidate genes was evaluated in a transient expression system in *N. benthamiana*. It has been reported that endogenous *N. benthamiana* enzymes may derivatize oxidized monoterpenes to non-volatile glycosides (Dong *et al.*, 2016). To assess if conversion products are formed from the pathway intermediates, limonene, POH, PAldH, and PA were infiltrated in *N. benthamiana,* and leaves were analysed by GC-MS, to detect volatile products, and by LC-MS to detect non-volatile derivatives (Supplementary Tables S5, S6). The analysis showed that limonene is hardly modified by this plant platform, and was emitted into the leaf headspace. Other pathway intermediates, including POH, PAldH, and PA, are derivatized to non-volatile products. Based on these results, we further selected marker compounds as proxy for the presence of the pathway products in *N. benthamiana*. The markers were putatively identified on an MS/MS spectrum basis as perillyl alcohol malonyl hexose (POH-Mk1), perillyl alcohol pentose-hexose (POH-Mk2), a perillyl aldehyde gluthathione conjugate (PAldH-Mk1), perillic acid hexose (PA-Mk1), hydroxylated perillic acid hexose (PA-Mk2), and perillic acid di-hexose (PA-Mk3) (Supplementary Table S5). These markers were used to monitor the formation of the pathway intermediates in the plant, upon candidate gene expression.

### Cytochrome P450 SdL7H converts limonene to perillyl alcohol, but does not perform further oxidations

**Fig. 4.**
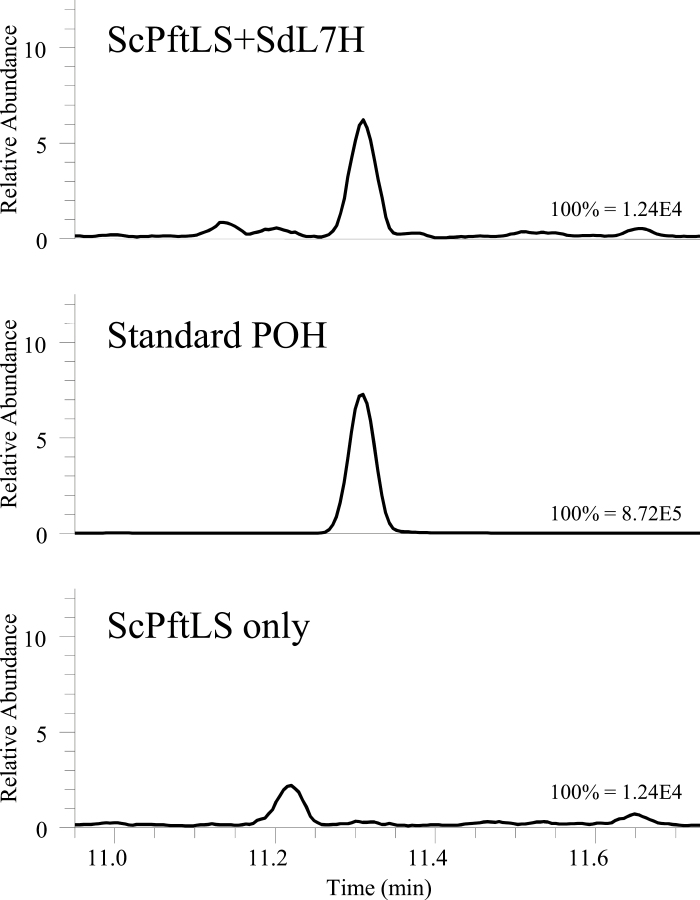
Characterization of SdL7H in yeast. Shown are GC-MS chromatograms of culture extracts. (a) Yeast strain containing limonene synthase and limonene-7-hydroxylase (ScPftLS+SdL7H) TIC NL 1.24E4 *m/z* 93–94. (b) Perillyl alcohol standard reference compound TIC NL 8.72E5 *m/z* 93–94. (c) Yeast strain containing only limonene synthase (ScPftLS) TIC NL 1.24E4 *m/z* 93–94. Mass spectra of the analysed peaks are shown in Supplementary Fig. S8.

Six cytochrome P450 candidate sequences (*CYP71A76v1*, *CYP71A76v2*, *SdCYP76AH36*, *SdCYP76AK17*, *SdCYP71A77*, and *SdCYP76AK18*) were cloned for characterization (Supplementary Fig. S8). SdCYP71A76v1 and SdCYP71A76v2 differ in only two C-terminal amino acids (Supplementary Fig. S8) and did not differ in activity. In yeast co-expressing *SdCYP71A76v1* (referred to as *SdL7H*) with LS, formation of POH was observed (Fig. 4; Supplementary Fig. S9), while no PAldH or products of oxidation of limonene on other positions were detected.

*SdL7H* was also cloned for characterization *in planta.* For comparison, *MsL3H* (Lücker *et al.*, 2002) was also cloned in the same system. Both constructs were co-infiltrated with *SdLS* in *N. benthamiana* (Supplementary Fig. S10), to provide limonene as a substrate. GC-MS analysis showed there was a small amount of POH in the headspace of the *N. benthamiana* leaves infiltrated with *SdL7H* and *SdLS* (Supplementary Fig. S10a–c). LC-MS analysis showed the presence of the POH-Mk1 and Mk2 markers in the leaves (Supplementary Fig. S10d). In addition, expression of *SdL7H* resulted in a decrease in the amount of limonene in the headspace, indicating that limonene is indeed used as a substrate (Supplementary Fig. S10e).

Enzymes which were candidates for conversion of POH to more downstream products were tested in *N. benthamiana* immediately, allowing their performance in a plant background to be addressed. LC-MS was used to analyse the enzyme products (PAldh and PA), since these compounds are immediately further derivatized by *N. benthamiana*. An alcohol dehydrogenase gene referred to as *SdPOHDH* was isolated from cDNA, together with four other candidates (Supplementary Fig. S11). All genes were cloned for testing in *N. benthamiana*, and were agroinfiltrated with POH as a substrate. LC-MS analysis of POH and PAldH markers showed that SdPOHDH converted POH to PAldH (Supplementary Fig. S12). For the other candidate dehydrogenase, no such activity could be observed.

### SdPAOMT methylates both perillic acid and phenolic acids into volatile methyl esters

Several methyltransferase (OMT) sequences (Supplementary Fig. S13) were cloned for characterization and co-infiltrated in *N. benthamiana* with PA. For one candidate gene, which was referred to as *SdPAOMT*, a significant amount of MePA was found in the headspace of the agroinfiltrated leaves, while only SdOMT4 and SdOMT9 showed minor MePA formation, and the other candidates were not different from the control (Supplementary Fig. S14a, b). Although *SdPAOMT* was not the most highly expressed OMT gene in trichomes, we reasoned that it could mediate formation of MePA in *S. dorisiana*.

The protein sequence of SdPAOMT was aligned using BLASTP analysis, and was found to be similar to methyltransferases from the SABATH family (Supplementary Fig. S13a). This family includes enzymes involved in methylation of carboxyl groups of aromatic compounds or methylating nitrogen groups (D’Auria *et al.*, 2003). The most closely related homologs of SdPAOMT include a number of enzymes that are involved in methylation of BA and/or SA (Supplementary Fig. S13), for example AmSAMT, encoded by a gene from snapdragon involved in methylation of SA in vegetative tissues (Negre *et al.*, 2002).

The ability of SdPAOMT to methylate SA, BA, and PA was tested *in vitro*. To this end, the *SdPAOMT* cDNA was cloned in the expression vector pCDF DUET-1, and SdPAOMT protein was produced in *E. coli* BL21 DE3. Cell-free extracts were tested with BA, SA, and PA as substrates, and SAM as methyl donor. Methylated products were detected for all three substrates (Fig. 6). In contrast, methylation of JA could not be observed. This indicated that *in vitro* SdPAOMT is able to methylate both phenolic and monoterpenoid carboxylic acids.

**Fig. 5.**
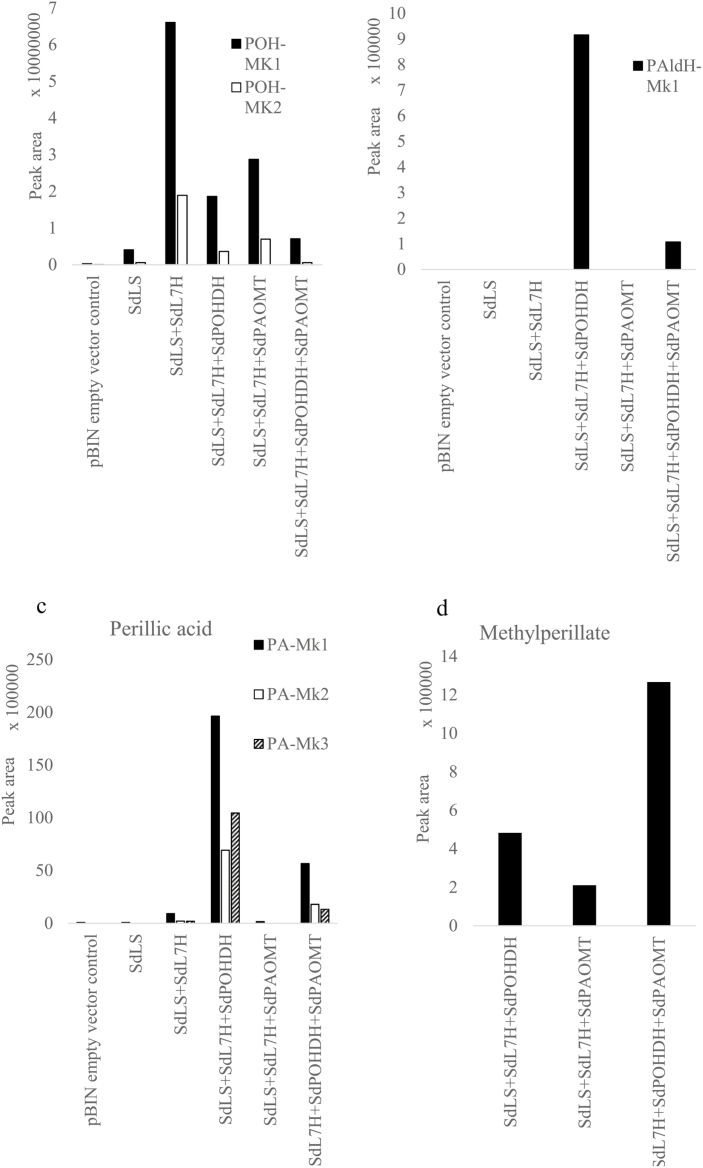
Reconstitution of the methylperillate biosynthesis pathway in *N. benthamiana*. Shown are relative quantities of marker compounds, representing intermediates in the methylperillate pathway. (a) Perillyl alcohol; (b) perillyl aldehyde; (c) perillic acid; (d) methylperillate. MK: marker compound.

**Fig. 6.**
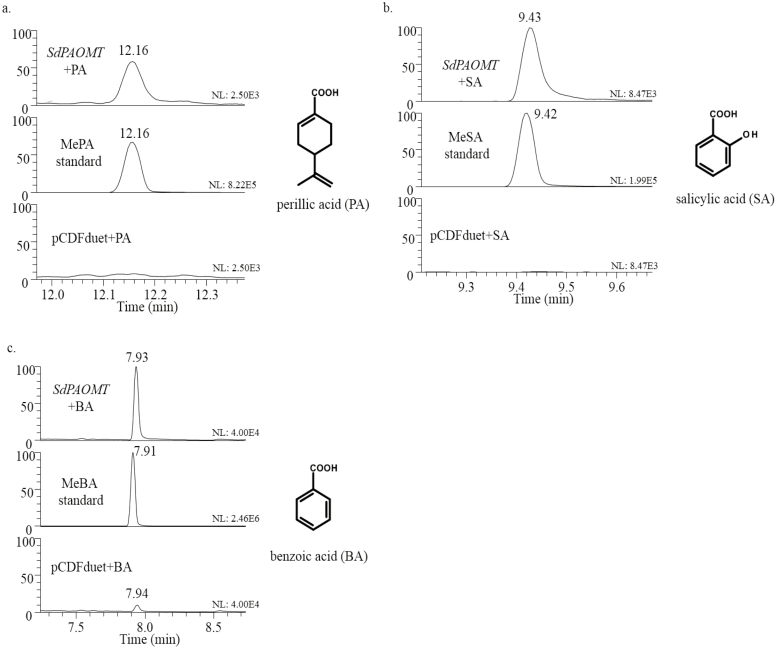
Characterization of *S. dorisiana O*-methyltransferase SdPAOMT. Shown are GC-MS chromatograms of *in vitro* assays with different substrates. (a) SdPAOMT+perillic acid (PA), *m/z*=67.50–68.50 + 120.50–121.50 + 179.50–180.50 MS. (b) SdPAOMT+salicylic acid (SA), *m/z*=151.50–152.50 MS. (c) SdPAOMT+benzoic acid (BA), *m/z* 76.50–77.50 + 104.50–105.50 + 135.50–136.50 MS. pCDFduet empty vector control.

To further investigate the activity of SdPAOMT, *N. benthamiana* leaves were infiltrated with a construct with the *SdPAOMT* cDNA. Five days after *SdPAOMT* infiltration, PA, BA, or SA were co-infiltrated and, after 3 h of incubation, the headspace was trapped for 3 h and analysed by GC-MS. Interestingly, infiltration of *SdPAOMT* resulted in emission of detectable amounts of both methylsalicylate (MeSA) and methylbenzoate (MeBA), alongside MePA (Supplementary Fig. S15). This confirms that SdPAOMT is also able to methylate both phenolic acids and monoterpene acids. In the headspace of *S. dorisiana* leaves, on the other hand, only traces of MeSA were observed, and no MeBA could be detected (Supplementary Fig. S15d), while MePA was one of the major detected peaks.

### Reconstitution of the methylperillate biosynthesis pathway in *N. benthamiana*

Subsequently, the full MePA biosynthesis pathway (Fig. 1) was transiently expressed in *N. benthamiana*, through agro-infiltration. Strains were co-infiltrated, each time adding a subsequent cDNA, while the load for each of the cDNAs was kept equal. While *N. benthamiana* can provide the necessary precursors from the MEP pathway, it is known that co-expression of an additional ectopic GPPS can boost production of monoterpenes in *N. benthamiana* (Dong *et al.*, 2016). Accordingly, co-expression of *Picea abies* GPPS (*PaGPPS*) with *SdLS* increased limonene emission 3- to 5-fold (Supplementary Fig. S16). Addition of *SdL7H* resulted in the production of POH, as observed from the appearance of peaks POH-Mk1 and POH-Mk2 analysed by LC-MS (Fig. 5a; Supplementary Fig. S19 panel c1–c2). Subsequent co-expression of *SdPOHDH* strongly decreased the peak area of these products, and resulted in the appearance of PAldH-Mk1 and PA-Mk1-3 (Fig. 5a–c; Supplementary Fig S19 panel d1–d6). GC-MS analysis showed that an easily detectable amount of PAldH was emitted (Supplementary Fig. S17). Interestingly, a small amount of MePA was detected from the headspace (Fig. 5d; Supplementary Fig. S19 panel d7), indicating that *N. benthamiana* expresses an endogenous methyltransferase able to methylate PA. Co-expressing *SdPAOMT* led to a strong increase of MePA emission (Fig. 5d; Supplementary Fig. S19, panel e7), while POH-Mk1-2, PAldH-Mk1, and PA-Mk1-3 peaks were strongly reduced (Fig. 5a–c; Supplementary Fig. S19, panel e1–e6). When *SdPAOMT* was co-expressed with *PaGPPS*, *SdLS*, and *SdL7H*, but without *SdPOHDH*, MePA emission was 6-fold lower (Fig. 5d; Supplementary Fig. S19), indicating the importance of *SdPOHDH* for the pathway.

Thus, *de novo* biosynthesis of MePA was achieved in *N. benthamiana* by expressing *PaGPPS*, *SdLS*, *SdL7H*, *SdPOHDH*, and *SdPAOMT* (Fig. 5; Supplementary Fig. S19, panel e7).

## Discussion

In the present study, the genes needed for biosynthesis of MePA have been identified. This compound is of interest, since it can be converted to the commodity chemical TA by mild chemistry (Jongedijk *et al.*, 2018). To this end, candidate genes from *S. dorisiana*, selected based on expression profiles, were identified and characterized *in vitro* and in *N. benthamiana*. From these screens, four genes could be identified which together constitute a biosynthetic pathway from GPP to MePA. Apart from well-known terpene synthases and terpene-modifying enzymes such as cytochrome P450s and alcohol dehydrogenases, a terpene carboxylic acid *O*-methyltransferase was identified. Together, the genes form a MePA biosynthesis pathway, although the current data do not exclude that other enzymes encoded in *S. dorisiana* play a role in the pathway to MePA. The genes identified here were sufficient to produce MePA in *N. benthamiana*. The identification of these enzymes provides a basis for a novel way to produce biomaterials not only from natural, renewable sources, but also with specific stereochemistry.

### Regio-specificity of SdL7H is essential for methylperillate formation

MePA carries an unusual functional group for a monoterpene, a methylated carboxyl group at the *para*-position, which is essential for its use as a precursor for TA synthesis (Jongedijk *et al.*, 2018). To use limonene as a direct precursor for TA, an enzyme that modifies limonene at its C7 position is essential, and SdL7H is capable of this reaction. After hydroxylation of the 7-position by SdL7H, the resulting primary alcohol group is available for further oxidation to the acid. This type of oxidation has been described for sesquiterpenes such as artemisinic alcohol, to form artemisinic acid, and germacrene A alcohol, to form germacrenoic acid (Ro *et al.*, 2006; Liu *et al.*, 2011). Several P450s acting on limonene or derived compounds have been identified in the *Mentha* genus (Bertea *et al.*, 2003; Lücker *et al.*, 2004). However, these enzymes display a different regio-specificity; they modify limonene on different carbon positions of the molecule. The secondary alcohols formed by mint P450s at the 3- and 6-position of limonene cannot be taken further towards carboxyl groups, and therefore these enzymes are not applicable for generating a polymer precursor such as TA. From *Perilla frutescens*, a cytochrome P450 has been described to produce a mixture of C3, C6, and C7 hydroxylated compounds (Mau *et al.*, 2010). This enzyme is likely to be involved in the formation of PAldH in *P. frutescens*, but it lacks the specificity required for application in an efficient heterologous production system. Recently, a more regio-specific enzyme from *Perilla* has been described (Fujiwara and Ito, 2017). This enzyme catalyses both the conversion of limonene to POH and the subsequent dehydrogenation to PAldH. SdL7H is highly regio-specific, as it produces PAldH as the single limonene hydroxylation product both in yeast and *in planta*. Like all other monoterpene P450s known to date (including the *Perilla* and *Mentha* genes), SdL7H is a member of the CYP71 class, but shares very limited sequence similarity with other CYP71s known to use limonene as substrate (e.g. *M. spicata* L6H, 37% identity; *M. spicata* L3H, 35% identity; *P. frutescens* L367H; 40% identity). *SdL7H* shares a high sequence identity (69%) with menthofuran synthase, a P450 from *Mentha×piperita* (Bertea *et al.*, 2001). Menthofuran synthase acts on an alyllic carbon in pulegone. Though this enzyme is not active on limonene, notably it does act on a non-ring position of a monoterpene and initially forms a primary alcohol group. This indicates that the formation of a primary alcohol may have been the property of the common ancestor of these enzymes.

*Nicotiana benthamiana* appears to have endogenous POHDH-, PAldHDH- and PAOMT-like activities, albeit at a low level. Therefore, it makes sense to apply specialized enzymes recruited from *S. dorisiana* for these conversions. This is clearly seen when *SdPOHDH* is added to the system, by which MPA production is enhanced 6-fold. The properties of SdPOHDH largely correspond to those described describe for a POHDH from *P. frutescens* (Sato-Matsumoto and Ito, 2014), although the amino acid sequences of both proteins are only 21.4% identical.

For conversion of PAldH to PA, no enzyme has yet been isolated from *S. dorisiana*. Several different enzyme classes have been reported to catalyse terpene aldehyde oxidation, such as cytochrome P450 enzymes. For example, the formation of deoxyloganetic acid from iridodial in *C. roseus* is performed by iridoid oxidase, which is a P450 (CYP76A26) (Miettinen *et al.*, 2014). On the other hand, artemisinic aldehyde is converted efficiently to artemisinic acid by a member of the aldehyde dehydrogenase family from *A. annua* (Teoh *et al.*, 2009). Both these enzyme classes were over-represented in the subset of genes from which MePA pathway genes were isolated (Supplementary Table S3). Formation of monoterpene acids has not yet been extensively investigated. In other plants such as Pyrethrum (*T. cinerariifolium*) and *C. roseus*, acid forms of monoterpenes play a role in transport between cells. However, in *S. dorisiana*, the free acid form could not be detected. Apparently *S. dorisiana* prevents PA being present in the free form by methylating it efficiently in the trichomes.

### Formation of a monoterpene methylester

Methylation of PA makes the compound volatile, which could be related to its biological function in *S. dorisiana*. The enzyme which mediates methylation of PA most efficiently *in planta*, SdPAOMT, has high similarity to methyltransferases from the SABATH family, which have been found to use phenolic compounds, such as BA and SA, as substrates. MeBA is part of the fragrance of flowers, for instance in *Clarkia brewerii*, where the formation of MeBA was suggested to result in the attraction of bumblebees for pollination (Kolosova *et al.*, 2001). Similarly, *SdPAOMT* may play a role in the formation of highly volatile scented compounds in the foliage of *S. dorisiana*. Methylation of SA was suggested to play a role in the modulation of the activity of SA, which functions as a defence signal. Methylation of SA facilitates transport from plant to plant due to its volatility and membrane permeability (Dempsey *et al.*, 2011). We show that SdPAOMT can also function as a BA-OMT and SA-OMT. In *S. dorisiana* trichomes, these substrates seem not to be relevant, as MeBA and MeSA are hardly detectable in the *S. dorisiana* headspace. With the current data, we cannot conclude that SdPAOMT is the only OMT mediating formation of MePA, as no knockout experiments have been included which could clarify the contributions of SdPAOMT in MePA formation in *S. dorisiana*. Also other OMTs are able to mediate methylation of PA (e.g. SdOMT9); however, their much lower efficiency in *Nicotiana* compared with SdPAOMT (Supplementary Fig. S14) suggests a key role for this enzyme in MePA formation. With this reservation, *SdPAOMT* may be considered to be a neo*-*functionalized gene that has been employed from pathways leading to more general phenolic volatiles, to participate in the production of a specialized monoterpene methylester.

*SdPAOMT* can play a key role in the engineering of monoterpene carboxylic acids in plants. When PA is produced in *N. benthamiana*, a number of glycosides are produced (Supplementary Table S6). Glycosylation probably aims at the detoxification of these compounds, allowing their sequestration to the vacuole. Methylation, on the other hand, makes the compound more hydrophobic and facilitates its diffusion out of the cell. In *P. frutescens*, PA is known to be stored as a glycoside. *SdPAOMT* expression decreases the formation of glycosides in *N. benthamiana*, indicating that *SdPAOMT* efficiently competes with glycosyl transferases from *N. benthamiana*. The mechanism involved in the modulation of PA toxicity through acid methylation still remains unclear, and the defence properties of these terpenes need to be investigated.

### Outlook

The next step for producing biobased commodity chemicals through this route is to produce MePA on a larger scale. Plant production may be suited for this in the longer term, but terpene production in microorganisms has been much further advanced (Jongedijk *et al.*, 2016). Limonene production has also been demonstrated in cyanobacteria, opening up the possibility to produce MePA, as a source for PET plastic from carbon dioxide and sunlight (Wang *et al.*, 2016). Alternatively, MePA biosynthetic enzymes could be expressed in microorganisms. Yeast strains producing limonene have been reported. In general, plant P450s can be expressed rather well in yeast (Arendt *et al.*, 2017), so this could provide a suitable platform for further engineering towards MePA production. In *Pseudomonas putida*, a bacterium with high solvent tolerance, reasonable amounts of PA were already produced by bioconversion of limonene, by means of side activity of the *p*-cymene degradation gene cluster (Mars *et al.*, 2001). By elucidating pathways towards MePA and other functionalized monoterpenes, engineering of these compounds in microorganisms should now be possible. From this perspective, the elucidation of the MePA pathway in *S. dorisiana* is the first step towards biobased production of commodity chemicals. Thus, this work provides a basis for a novel way to produce biomaterials from natural, renewable sources.

## Supplementary data

Supplementary data are available at *JXB* online.

Dataset S1. DNA sequences of selected clusters and genes.

Fig. S1. Concentrations of methylperillate and pathway intermediates in *S. dorisiana* plant parts.

Fig. S2. GC-MS chromatograms of *S. dorisiana* trichomes and young leaves on an enantioselective column.

Fig. S3. Isolated *S. dorisiana* trichomes.

Fig. S4. Separated *S. dorisiana* trichome fractions.

Fig. S5. Protein sequence alignment and phylogenetic tree of monoterpene synthases.

Fig. S6. GC-MS chromatograms of *N. benthamiana* headspace after agro-infiltration with limonene synthases.

Fig. S7. *In vitro* assay of other *S. dorisiana* monoterpene synthases.

Fig. S8. Protein sequence alignment of P450s.

Fig. S9: Characterization of *SdL7H* in yeast: MS spectra.

Fig. S10. Characterization of *SdL7H* in *N. benthamiana* after agro-infiltration of constructs.

Fig. S11. Alcohol dehydrogenases (ADH) protein sequence alignment and phylogenetic tree.

Fig. S12. Characterization of *SdPOHDH* activity.

Fig. S13. *O-*Methyltransferases protein sequence phylogenetic tree and expression levels of *S. dorisiana* methyltransferase candidates.

Fig. S14. *SdPAOMT* produces methylperillate in *N. benthamiana.*

Fig. S15. Headspace GC-MS analysis of *N. benthamiana* infiltrated with *SdPAOMT* and *S. dorisiana* leaves.

Fig. S16. Co-expression of GPP synthase (*GPPS*) increases limonene emission.

Fig. S17. Agro-infiltration of *SdPOHDH* produces perillyl aldehyde in the headspace of *N. benthamiana* leaves.

Fig. S18. Methylperillate in the headspace of agro-infiltrated *N. benthamiana* leaves.

Fig. S19. Chromatograms of LC-MS and GC-MS analyses of methylperillate intermediates and derivatives.

Table S1. Primer sequences.

Table S2. Relative expression data.

Table S3. Set of transcripts that had a high absolute expression level in the trichomes, a high correlation with presence of a pathway, and a high correlation with co-expression with *SdLS*.

Table S4. PFAM domains that were enriched in the extracted group of genes.

Table S5. LC-MS of marker compounds in *N. benthamiana.*

Table S6. Compound infiltration peak areas; identification of markers.

Table S7. Constructs used in this study.

eraa086_suppl_Supplementary_Tables_S1_S2_S4_S7

eraa086_suppl_Supplementary_Tables_S3

eraa086_suppl_Supplementary_Figures_S1_S19

eraa086_suppl_Supplementary_Dataset

## Abbreviations

BA: benzoic acid
GPP: geranyl diphosphate
GPPS: geranyl diphosphate synthase
L7H: limonene-7-hydroxyase
LS: limonene synthase
MEP: methyl-D-erythritol 4-phosphate
MePA: methylperillate
Mk: marker compound
PA: perillic acid
PAldH: perillyl aldehyde
PAOMT: perillic acid *O*-methyltransferase
PET: polyethylene terephthalate
POH: perillyl alcohol
POHDH: perillyl alcohol dehydrogenase
SA: salicylic acid
Sd: *Salvia dorisiana*
