# Supplementary material for: Novel routes towards bioplastics from plants: elucidation of the methylperillate biosynthesis pathway from Salvia dorisiana trichomes
Source: J Exp Bot. 2020 Feb 24;71(10):3052–65. doi: 10.1093/jxb/eraa086 (PMC7260718; doi:10.1093/jxb/eraa086)
Supplement: eraa086_suppl_Supplementary_Tables_S1_S2_S4_S7 [file eraa086_suppl_supplementary_tables_s1_s2_s4_s7.pdf]

Table S1 Primer sequences used in this study

primer*	reference	sequence
SdLS_NotF		TATGCGGCCGCATGTCTAGTATTATAATGCAATTCGCCATTCCTA
SdLS_PacR		ACTTTAATTAATTACTCATAAGGTTCGAACAGCAAGCTTCT
SdtLS_SalF		ACTGTCGACCGACGAACTGGAGGCTACCA
SdLS_NotR		TGCGGCCGCTTACTCATAAGGTTCGAACAGCAAGCTTCT
SdtPS_AscF		ATGGCGCGCCTGCGAAGATCGGGAAACTACAGTCCAT
SdPS_NotR		ATGCGGCCGCTCAGGCAATAGGGTGGAACAAGCAAT
SdtLiS_AscF		ATGGCGCGCCTGCGACGCTCCGGAAACTACCA
SdLiS_NotR		ATGCGGCCGCTCAATCACATGGTTGAAACAGCAGACT
SdtPCS_AscF		ATGGCGCGCCTGCGACGGTCGGGTAATTACAAGCCTA
SdPCS_NotR		ATGCGGCCGCCTAGACAATAGGTTGAAATATCAAACCTAAAATACGTT
SdtSaS_AscF		ATGGCGCGCCTGAGGAGGTCTGCAAACTACGAAGCTAGT
SdSaS_NotR		ATGCGGCCGCTCAAGGCATCGAAAAGGGCTGAA
SdtEuS_AscF		ATGGCGCGCCTGCGACGAACTGGAGGCTACCAGCCTACT
SdEuS_NotR		ATGCGGCCGCTTATTCATAAGGTTCGAACAGTAAGCTTCTGAGCTGTT
ClLS_NotF	(Lücker et al., 2002)	TAGCGGCCGCTATGTCTTCTTGCATTAATCCCTCAACCTTGGTTACCT
ClLS_PacR	(Lücker et al., 2002)	ACTTAATTAATCAGCCTTTGGTGCCAGGAGATGC
PfLS_NotF	(Jongedijk et al., 2015)	TAGCGGCCGCTATGTATACCGGTGTAATAATGCATATGGCGATTCCT
PfLS_PacR	(Jongedijk et al., 2015)	ACTTAATTAATTACAACCATTGCTCGAACAAGATGTCTGTCATCT
SdL7H_GWF		CACCATGGCAGCTCTTCTACTTCTTA
SdL7H_R		TTAATAAGCACGTGGTGTAGTAA
SdADH_GWF		CACCATGGCTGATAACACCATAACTT
SdADH_R		TCAGAACTTGATAATGACCTTGA
SdOMT_BamF		TACGGATCCGATGGAAGTAGTTGAGGTGCTTC
SdOMT_NotR		AATGCGGCCGCTCATCCTCTCCGGATCAAA

RT2_R	GAAAGAGATAAGAAGTAGAAGAGCTGCCATACT AGTTCTAGAAAACTTAGATTAGATTGC
RT3_F	GCAATCTAATCTAAGTTTTCTAGAACTAGTATGG CAGCTCTTCTACTTCTTATC
RT4_R	AAACAAGAATCTTTTTATTGTCCTCGAGAATTAA TAAGCACGTGGTGTAGTAACC
RT5_F	CTTGTGGTTACTACACCACGTGCTTATTAATTCTC GAGGACAATAAAAAGATTCTTG
RT6_R	AGACAGATCGCTGAGATAGG
RT7_F	CTCACGTTAAGGGATTTTGGTC
RT8_R	TAATTGGTGCATCAGGTGGTCATGGCCCTTCGTT AAAGGACAAGGACCTGA
RT9_F	CACTTCCGCTCAGGTCCTTGTCCTTTAACGAAGG GCCATGACCACCTGA
RT13_F	TAAGAAAGCAACACCTGGCAATTCCTTACCACTA GTTCTAGAATCCGTCGA
RT14_R	AACTTAGTTTCGACGGATTCTAGAACTAGTGGTA AGGAATTGCCAGGTGTTGC

***F: forward; R: reverse; t: truncated, removes plastid targeting signal; Sal: SalI restriction site added, Asc: AscI restriction site added; Not: NotI restriction site added; GW: cacc tag added for directional cloning in pENTR/D-TOPO.**

Supplemental Table S2 Sequencing data HiSeq sequencing

Sample ID	Index	# Reads	% of raw clusters	% of >= Q30 Bases (PF)	Mean Quality Score (PF)
Leaves >10 cm	CGTACG	74248274	13.30	95.13	35.79
Leaves3-10 cm	GTTTCG	149802556	26.83	95.07	35.77
Leaves < 3 cm	GTGGCC	106516624	19.08	94.82	35.72
Roots	ACTGAT	57144448	10.23	95.25	35.80
Stem	GAGTGG	59690696	10.69	95.18	35.80
Trichomes	ATTCCT	38229990	6.85	94.76	35.62
Undetermined	-	72713238	13.02	90.54	34.70
Total		279172913			

Supplemental Table S4. PFAM domains that were enriched in the extracted group of genes (correlation with the monoterpene synthase expression R>0.6, correlation with pathway metabolites pattern R>0.6 and expression in the trichomes RPKM>1000), compared to all other genes.

PFAM domain	p-value	description
p450	0.000	P450
Vir_act_alpha_C	0.000	a bacterial motif?
RPE65	0.000	Retinal pigment epithelial membrane protein / carotenoid oxygenase
ketoacyl-synt	0.001	Beta-ketoacyl synthase, N-terminal domain
Ketoacyl-synt_C	0.001	Beta-ketoacyl synthase, C-terminal domain, involved in fatty acid synthase
Thiolase_N	0.015	thiolase, N-terminal domain, acetyl-coenzyme A acetyltransferases (ACAT), are enzymes which convert two units of acetyl-CoA to acetoacetyl CoA in the mevalonate pathway.
DUF1949	0.020	a green algae motif? (domain unknown function)
DUF3106	0.020	a bacterial/nematode motif?
OSCP	0.023	ATP synthase delta (OSCP) subunit
Terpene_synth	0.025	Terpene synthase, N-terminal domain
Terpene_synth_C	0.040	Terpene synthase family, metal binding domain (with DDXXD)
DUF3353	0.040	a plant motif
EamA	0.040	EamA-like transporter family, EamA (named after the O-acetyl-serine/cysteine export gene in E. coli) is a protein domain found in a wide range of proteins including the Erwinia chrysanthemi PecM protein, which is involved in pectinase, cellulase and blue pigment regulation, the Salmonella typhimurium PagO protein (function unknown), and some members of the solute carrier family group 35 (SLC35) nucleoside-sugar transporters. Many members of this family have no known function and are predicted to be integral membrane proteins
EmrE	0.040	Putative multidrug resistance efflux transporter
TPT	0.040	Triose-phosphate Transporter family
UAA	0.040	UAA transporter family, This family includes transporters with a specificity for UDP-N-acetylglucosamine

Chalcone	0.040	Chalcone-flavanone isomerase
DUF3298	0.040	a bacterial/nematode motif?
Transferase	0.040	This family includes a number of transferase enzymes. These include anthranilate N-hydroxycinnamoyl/benzoyltransferase that catalyses the first committed reaction of phytoalexin biosynthesis [1]. Deacetylvindoline 4-O-acetyltransferase EC:2.3.1.107 catalyses the last step in vindoline biosynthesis is also a member of this family [2]. The motif HXXXD is probably part of the active site. The family also includes trichothecene 3-O-acetyltransferase.
DUF1295	0.040	a plant motif
ICMT	0.040	Isoprenylcysteine carboxyl methyltransferase (ICMT) family
PEMT	0.040	Phospholipid methyltransferase
Steroid_dh	0.040	3-oxo-5-alpha-steroid 4-dehydrogenase
BURP	0.049	BURP domain, It is found in the C-terminal part of a number of plant cell wall proteins
adh_short	0.049	The short-chain dehydrogenases/reductases family (SDR)[2] is a very large family of enzymes, most of which are known to be NAD- or NADP-dependent oxidoreductases. As the first member of this family to be characterised was Drosophila alcohol dehydrogenase, this family used to be called[3][4][5] 'insect-type', or 'short-chain' alcohol dehydrogenases. Most members of this family are proteins of about 250 to 300 amino acid residues. Most dehydrogenases possess at least 2 domains,[6] the first binding the coenzyme, often NAD, and the second binding the substrate.
adh_short_C2	0.049	Enoyl-(Acyl carrier protein) reductase
Epimerase	0.049	NAD dependent epimerase/dehydratase family, NADH dehydrogenase (ubiquinone)
KR	0.049	This enzymatic domain is part of bacterial polyketide synthases and catalyses the first step in the reductive modification of the beta-carbonyl centres in the growing polyketide chain. It uses NADPH to reduce the keto group to a hydroxy group
NAD_binding_10	0.049	NAD(P)H-binding
HD_assoc	0.049	Phosphohydrolase-associated domain, This domain is found on bacterial and archaeal metal-dependent phosphohydrolases
FA_hydroxylase	0.049	Fatty acid hydroxylase superfamily, This superfamily includes fatty acid and carotene hydroxylases and sterol desaturases. Beta-

		carotene hydroxylase is involved in zeaxanthin synthesis by hydroxylating beta-carotene, but the enzyme may be involved in other pathways [1]. This family includes C-5 sterol desaturase and C-4 sterol methyl oxidase. Members of this family are involved in cholesterol biosynthesis and biosynthesis a plant cuticular wax. These enzymes contain two copies of a HXHH motif. Members of this family are integral membrane proteins.
Wax2_C	0.049	WAX2 C-terminal domain
Dimerisation	0.049	dimerisation domain, This domain is found at the N-terminus of a variety of plant O-methyltransferases. It has been shown to mediate dimerisation of these proteins
Lipase_2	0.049	Lipase (class 2), This family consists of hypothetical C. elegans proteins and lipases. Lipases or triacylglycerol acylhydrolases hydrolyse ester bonds in triacylglycerol giving diacylglycerol, monoacylglycerol, glycerol and free fatty acids
Methyltransf_18	0.049	Methyltransf_18 (PF12847)
Methyltransf_2	0.049	O-methyltransferase, This family includes a range of O-methyltransferases. These enzymes utilise S-adenosyl methionine.
Methyltransf_25	0.049	Methyltransf_25 (PF13649)

Supplemental Table S5. LC-MS marker compounds in *N. benthamiana* of perillyl alcohol (POH-Mk1, POH-Mk2), perillyl aldehyde (PAldH-Mk1) and perillic acid (PA-Mk1, PA-Mk2, PA-Mk3), MS/MS fragments and putative identification.

**Marker**	**Retention time (min)**	***m/z***	**Molecular formula**	**Accurate mass**	**MS/MS fragments**	**Putative identification**
POH-Mk1	41.60	401.1815	C19H29O9	401.1812 [M+FA-H]	355.1763 [M-CO_2-H]<br>313.1657 [M-malonyl-H]	Perillyl alcohol malonyl hexose
POH-Mk2	32.29	491.21378	$C_{22}H_{35}O_{12}$	491.21290 [M+FA-H]	445.2087 [M-H]<br>313.1657 [M-Pent-H]<br>149.0465 [Pent-H]	Perillyl alcohol pentose-hexose
PAldH-Mk1	27.73	458.1967	C20H32O7N3S	458.1961 [M-H]	Not measured	Perillyl aldehyde glutathione conjugate
PA-Mk1	34.68	373.15042	$C_{17}H_{25}O_9$	373.14990 [M+FA-H]	327.14 [M-H]<br>165.09 [M-Hex-H]<br>161.05 [Hex-H_2O-H]	Perillic acid hexose
PA-Mk2	16.41	389.14551	$C_{17}H_{25}O_{10}$	389.14480 [M+FA-H]	343.1397 [M-H]<br>181.0870 [M-Hex-H]	Hydroxylated perillic acid hexose
PA-Mk3	27.83	535.20325	$C_{23}H_{35}O_{14}$	535.20270 [M+FA-H]	489.1982 [M-H]<br>327.1451 [M-Hex-H]<br>179.0562 [Hex-H]	Perillic acid di-hexose

Supplemental Table S6. Compound infiltration peak areas, identification of markers

Peak no	Retention time (min)	*m/z*	Marker name	Peak area, 5h after infiltration				
				control	Lim	POH	PALDH	PA
1	4.28	249.1244		2107358	28513273	5337966	21120907	4691719
2	10.02	472.2445		0	7974610	14343	2488523	22439
3	10.38	470.2291		0	47087256	1402301	22220249	1475851
4	11.8	470.2292		0	41740868	1432016	22991464	1365174
5	13.56	389.1449		0	0	1464512	1208158	2001543
6	15.08	373.1498		219784	4068115	1241129	2140030	505167
7	16.42	389.1451	PA-Mk2	0	17129	6174730	5399178	6688770
8	19.8	377.1815		5562242	28477575	13461486	16391137	7471055
9	20.74	355.14		184358	249540	253035	207218	382554
10	22.12	245.0929		742160	8988829	2685759	5614797	1624532
11	22.25	697.26		0	14700	4199	4901	98825
12	25.65	515.12		977333	3741553	2680293	2121002	868190
13	25.82	417.1764		1009170	11875602	3582632	4725641	2824189
14	27.3	303.1446		432789	6374311	407462	1793914	1294001
15	27.78	417.18		0	0	477351	7928681	0
16	27.84	535.203	PA-Mk3	0	0	49965	47615	2589553
17	27.9	193.0503		828049	3074491	3245854	2310747	2589
18	28.11	458.196	PAldH-Mk1					
19	30.3	521.22		0	0	2568257	349712	0
20	30.79	505.1923		2860	3804	270446	102881	827959
21	32.33	491.21	POH-Mk2	92354	158554	6701095	1842464	34388
22	34.72	373.1504	PA-Mk1	14396	65722	20441272	14270127	65382552
23	35.24	493.2282		274826	2719455	2422599	1532171	275016
24	35.54	359.17		9927	67937	9534065	1873426	1838
25	37.3	587.3066		110481	6157920	1534457	2354536	1233754
26	37.87	745.3278		74444	3074684	777210	1776991	675635
27	40.6	1023.4638		144220	6121419	1386684	2652541	1531017
28	41.27	369.1554		0	0	242778	247709	2341384
29	41.6	355.1757	POH-Mk1	391371	558622	43657681	19359350	163148
30	42	817.3492		30884	5350549	667303	1676738	867122
31	42.58	1029.5271		56784	9706766	1303004	3715206	1877211
32	43.87	357.1916		637038	36894123	11842116	17914491	3996974
33	46.83	967.4747		7543518	314104760	162575188	213923292	28245418
34	47.95	801.3557		907203	41618044	11528937	27683106	8903091
35	49.91	329.18		1687813	2257293	12488540	3761940	22779

Supplemental Table S6 (continued)

**Peak no**	**Retention time (min)**	***m/z***	**Marker name**	**Peak area, 72h after infiltration**				
				control	Lim	POH	PALDH	PA
1	4.28	249.1244		2529351	2283095	2620660	2680162	1487909
2	10.02	472.2445		0	0	2457	0	1923
3	10.38	470.2291		0	0	82301	0	11863
4	11.8	470.2292		0	0	82742	0	12829
5	13.56	389.1449		0	0	872931	1453231	5587923
6	15.08	373.1498		398430	325827	986369	455836	202784
7	16.42	389.1451	PA-Mk2	1	19734	3381477	5151410	15043420
8	19.8	377.1815		5994955	5683369	8721929	5660064	3961581
9	20.74	355.14		151663	125935	516227	737978	7795048
10	22.12	245.0929		658029	1845477	859264	1168495	1343070
11	22.25	697.26		0	0	0	23944	6894950
12	25.65	515.12		865325	821250	858626	1055721	648767
13	25.82	417.1764		1161285	988082	1987724	1138805	1431252
14	27.3	303.1446		486323	989419	535879	443165	484495
15	27.78	417.18		0	0	63747	1698844	0
16	27.84	535.203	PA-Mk3	0	0	151947	643246	17886532
17	27.9	193.0503		993954	1195313	1152301	509459	47835
18	28.11	458.196	PAldH-Mk1					
19	30.3	521.22		0	16683	1336455	755656	5561
20	30.79	505.1923		0	11195	707427	1101647	7532441
21	32.33	491.21	POH-Mk2	36265	358307	2524334	1190693	41008
22	34.72	373.1504	PA-Mk1	0	0	653804	1514932	10762005
23	35.24	493.2282		58171	53504	162212	64529	44829
24	35.54	359.17		3608	41937	1827179	644512	4692
25	37.3	587.3066		6900	14565	239027	17748	270276
26	37.87	745.3278		6220	15598	251869	22923	209165
27	40.6	1023.4638		17172	28245	336259	33825	698749
28	41.27	369.1554		0	5608	747985	1920920	5720947
29	41.6	355.1757	POH-Mk1	227864	1302040	28239079	16998899	282391
30	42	817.3492		6319	4937	84500	5386	147278
31	42.58	1029.5271		19572	42666	475387	29970	701090
32	43.87	357.1916		238971	404214	1303264	399457	476287
33	46.83	967.4747		680885	1583765	19724140	1123546	4903886
34	47.95	801.3557		0	0	4857318	0	1512461
35	49.91	329.18		303989	379631	484157	447848	0

Supplemental Table S7 Plasmids and strains used in this study

plasmid/strain	Antibiotic resistance	features
plasmids		
pCDF-duet	spectinomycin 100 µg / mL	T7 promoter
Impact pIV1A-1.1	gentamycin 20 µg / mL	Rubisco promoter and terminator
ImpacTim	gentamycin 20 µg / mL	Rubisco promoter and terminator, NotI/PacI restriction sites added
pBin+	kanamycin 50 µg / mL	
pENTR/D-TOPO	kanamycin 50 µg / mL	
pB7WG2	spectinomycin 100 µg / mL	35S promoter and terminator
pBIN+PaGPPS	kanamycin 50 µg / mL	Rubisco promoter and terminator, GPP synthase (Dong et al., 2016)
pTEF1p-SdL7H-Tef1t	ampicillin 100 µg / mL	ScTEF1 promoter and terminator, *SdL7H*, URA3 marker, 2mµ ori, *E. coli* AmpR marker and pBR322 ori
strains		
Escherichia coli BL21-CodonPlus® (DE3)-ril	chloramphenicol 100 µg / mL	(Kleber-Janke and Becker, 2000)
Agrobacterium tumefaciens Agl0	rifampicilin 40 µg / mL	(Lazo et al., 1991)
Saccharomyces cerevisiae Sc-PftLS		(Jongedijk et al., 2015)
Saccharomyces cerevisiae Sc-PftLS-SdL7H		*Sc-PftLS* containing pTEF1p-SdL7H-Tef1t
