# Supplementary material for: Novel routes towards bioplastics from plants: elucidation of the methylperillate biosynthesis pathway from Salvia dorisiana trichomes
Source: J Exp Bot. 2020 Feb 24;71(10):3052–65. doi: 10.1093/jxb/eraa086 (PMC7260718; doi:10.1093/jxb/eraa086)

Figure S1 Concentrations of methylperillate and pathway intermediates in *Salvia dorisiana* plant parts. Highest concentrations were found in the small leaves. Error bars indicate standard deviations (n=3).

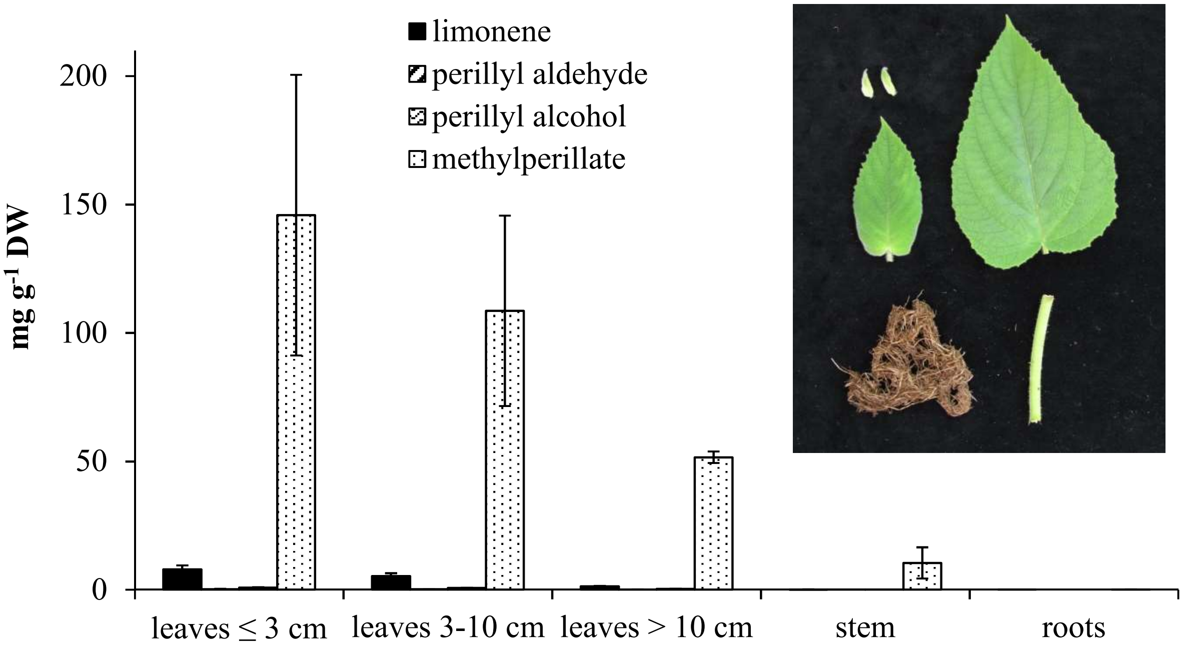

Fig. S2 GC-MS chromatograms on enantioselective column. *Salvia dorisiana* trichomes and young leaves contain exclusively the (-)-limonene enantiomer. m/z 68-69+136-137. RT 10.32-10.37 (-)-limonene, RT 11.22-11.25 (+)-limonene, RT 11.37-11.39 phellandrene peak visible in *Salvia dorisiana* chromatograms. Phellandrene was identified by its mass spectrum. In both (-)- and (+)-limonene standards a minor contamination from the other enantiomer is visible.

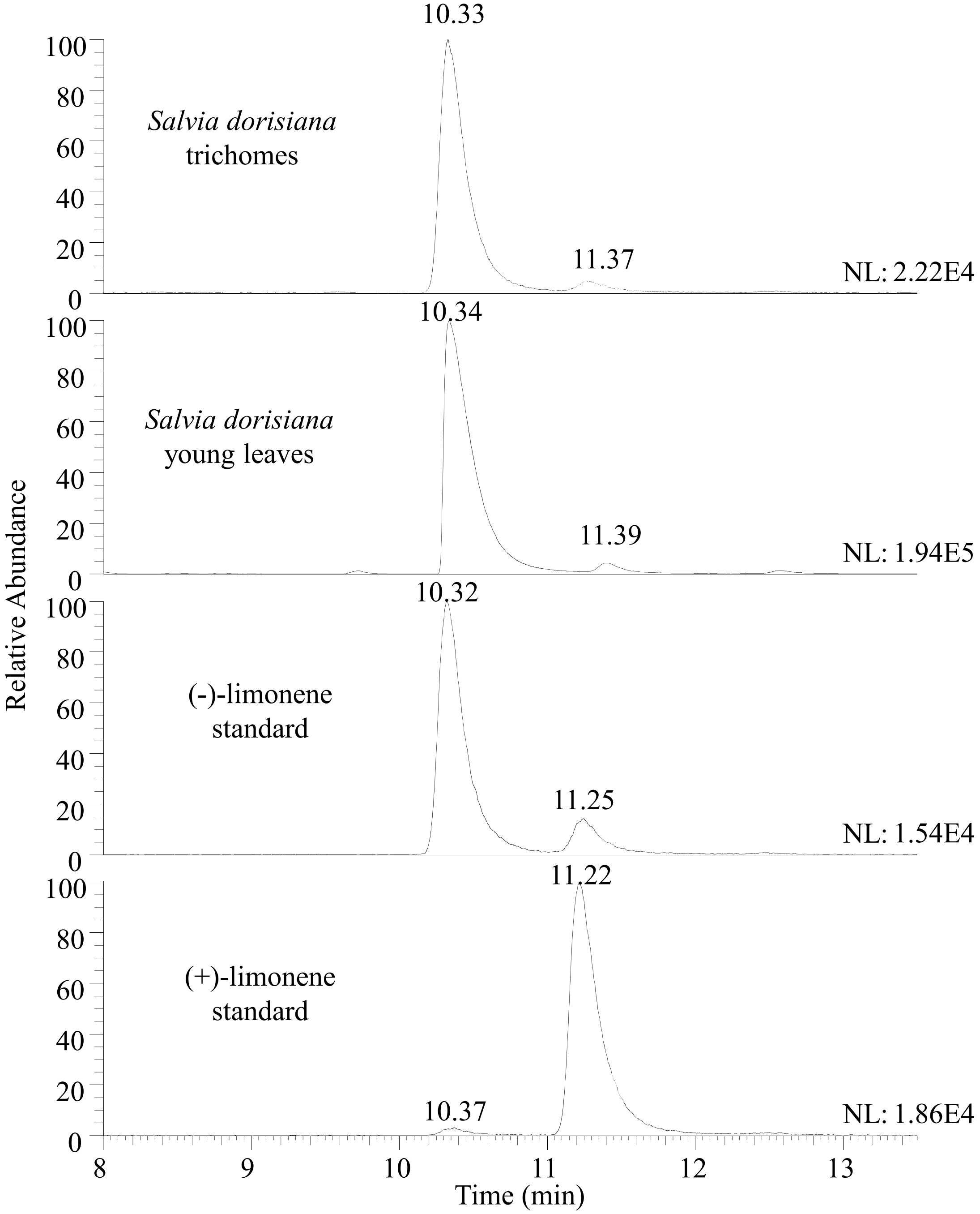

Fig. S3 Isolated S. dorisiana trichomes. a. light microscopy analysis, b. GC-MS chromatogram of isolated trichomes TIC NL 2.90E7. Peak annotations are provided below. Peaks have been identified by their MS spectrum by comparison to the NIST library. Peaks 10, 25, 26, 32 have additionally been identified by comparison to original standards.

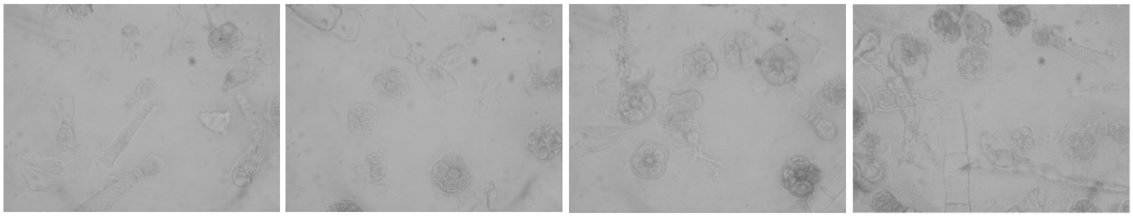

a.

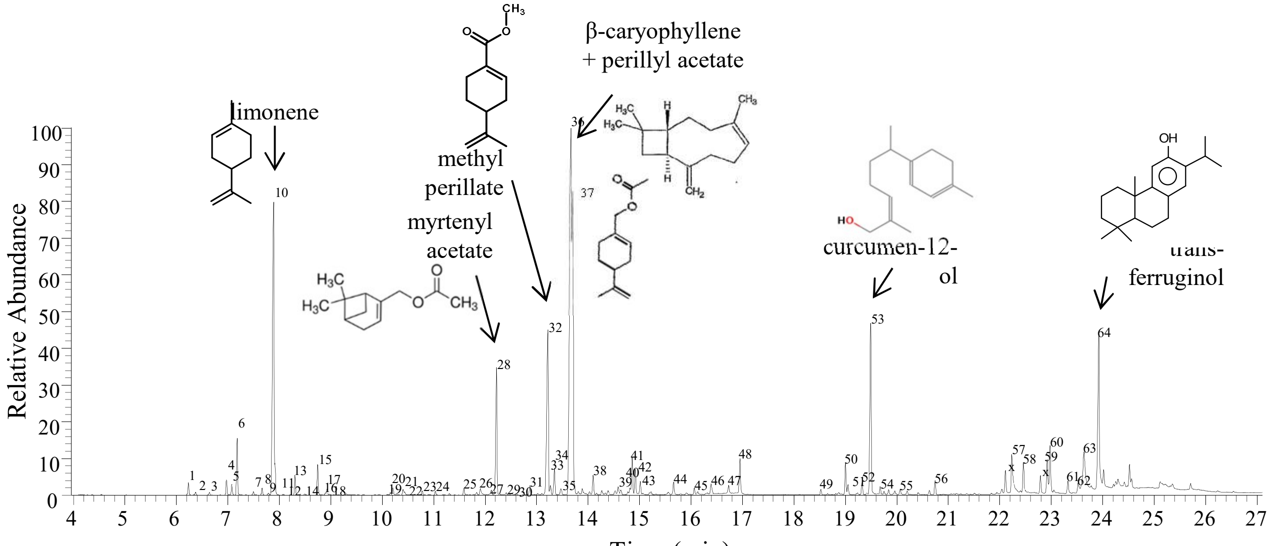

b.

1 α-thujene, 2 α-pinene, 3 camphene, 4 sabinene, 5 β-pinene, 6 myrcene, 7 α-phellandrene, 8 α-terpinene, 9 p-mentha-1,3,8-triene, 10 limonene, 11 trans-B-ocimene, 12 2-methylbutyl butanoate, 13 y-terpinene, 14 trans-4-thujanol, 15 terpinolene, 16 linalool, 17 butanoic acid, 2-methyl-, 2-methylbutyl ester, 18 cis-4-thujanol, 19 isopinocamphone, 20 terpinen-4-ol (internal standard), 21 a-terpineol, 22 8,9-epoxy limonene, 23 B-cyclocitral, 24 p-mentha-1,8-dien-10-ol, 25 perillyl aldehyde, 26 perillyl alcohol, 27 methylgeranate, 28 myrtenyl acetate, 29 bicycloelemene, 30 a-copaene, 31 geraniol acetate, 32 methyl perillate, 33 3-carene, 34 limonene-10-ol, 35 a-gurjunene, 36 B-caryophyllene, 37 perillyl acetate, 38 a-humulene, 39 dicyclogermacrene, 40 d-amorphene, 41 a-thujopsan-2-ol, 42 farnesol, 43 a-bisabolene, 44 caryophyllene oxide, 45 limonene-10-ol, 46 a-longipinene, 47 a-bisabolol, 48 6-A-hydroxy-germacra-1(10),4-dienepropanolyl-1H-inene ate, 49 1,6,10,14-Hexadecatetraen-3-ol, 3,7,11,15-tetramethyl-, (E,E)-, 50 trans-bergamota-2,12-dien-14-ol, (E)-, 51 1,6,10,14-Hexadecatetraen-3-ol, 3,7,11,15-tetramethyl-, (E,E)-, 52 9(11),15-isopimaradiene, 53 Z-y-curcumen-12-ol, 54 1,6,10,14-Hexadecatetraen-3-ol, 3,7,11,15-tetramethyl-, (E,E)-, 55 thujopsene, 56 16-kaurene, 57 diterpene, 58 4,5,6,7-Tetrahydroxy-1,8,8,9-tetramethyl-8,9-dihydrophenaleno[1,2-b]furan-3-one, 59 Podocarpa-1,8,11,13-tetraen-3-one, 14-isopropyl-1,13-dimethoxy-, 60 cis-ferruginol, 61 retinoic acid, 62 carnosol, 63 carnosic acid, 64 trans-ferruginol, x background (column material)

Figure S4 Separated *Salvia dorisiana* trichome fractions

Fresh *Salvia dorisiana* leaves of intermediate size were harvested (1-10 cm), and trichomes were isolated as described previously (Sallets et al. 2014), with following modifications: cutted leaves were shaken for 20 min 115 rpm. Trichomes were separated on size on different meshes, and flushed with washing buffer (Sallets et al. 2014): 350 um, 100 um, washed (100 um fraction was harvested: “type III and IV fraction”), 75 um and 45 um, and washed. The 45 um fraction was further separated by Percoll layers 10-30-60-80%, trichomes were harvested from the 30-60% interface and loaded on a 10-60% continuous sucrose gradient, trichome layer was visible in the light and harvested “type VI fraction”.

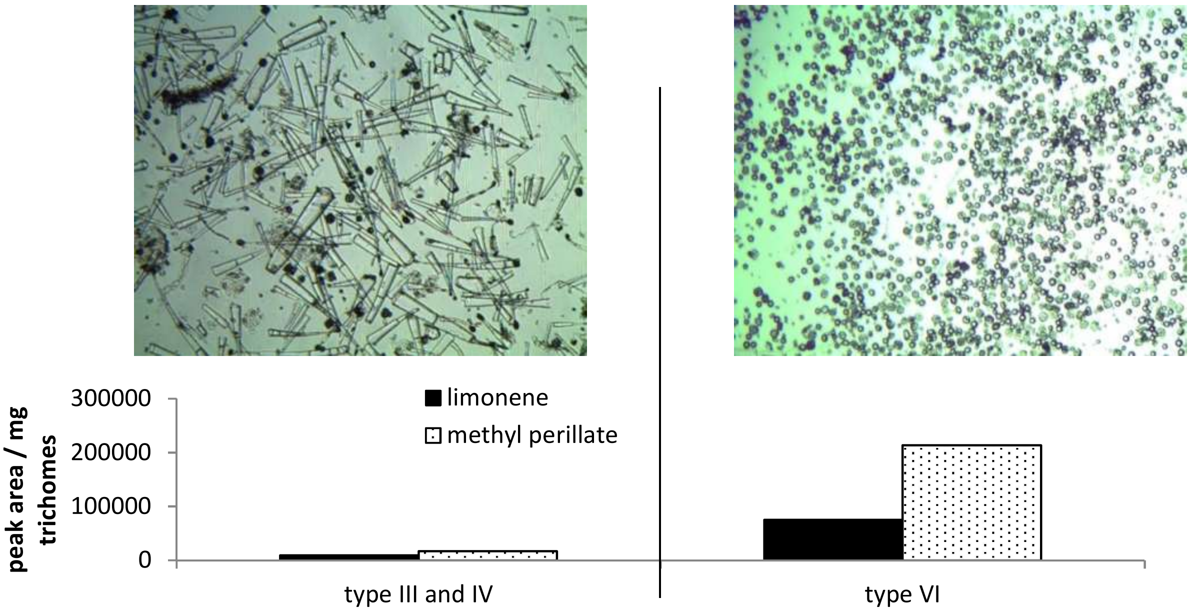

Figure S5 Protein sequence alignment and phylogenetic tree of monoterpene synthases. Colored lines indicate typical monoterpene synthase elements: green: plastid targeting signal (absent in SdSaS which has an in-frame stop codon 3 amino acids upstream of Met in the 5'UTR), orange RRX$_8$W motif and blue DDXXD metal binding motif

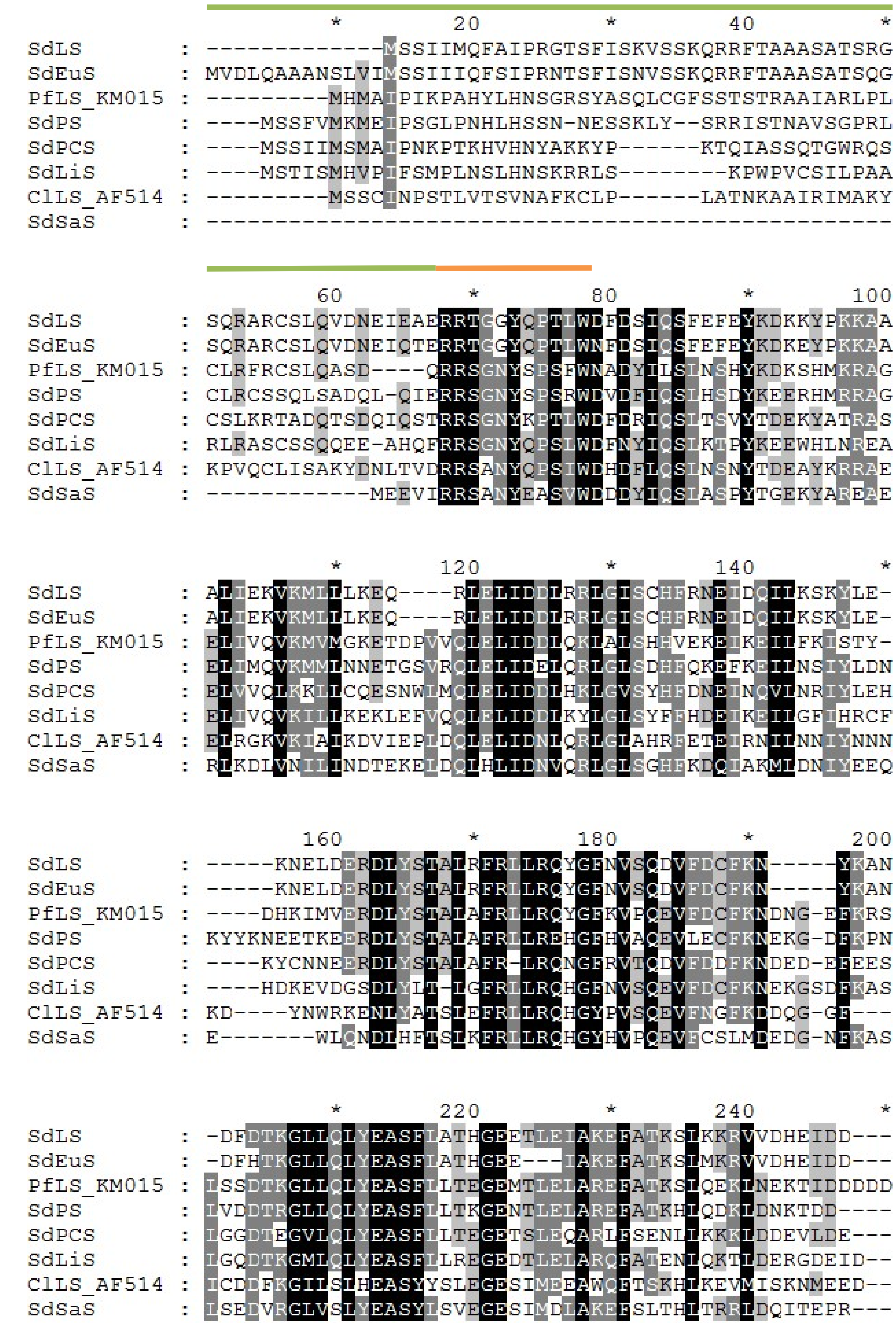

```
                          260         *       280         *       300
SdLS        : --IHFLSSVESALEFPSHWRVQMPYAKSFIDAYKNRQDMNPVVLELAILD
SdEuS       : --IHLLSSVEAALEFPSQWRVQMPNAKSFIDAYKRRPDMNPVVLELAILD
PfLS_KM015  : ADTNLISCVRHSLDIPIHWRIQRPNASWWIDAYKRRSHMNPLVLELAKLD
SdPS        : ---NLSTWIQYSLDIPIHWRIQRANASMWINAYKERPDMDPVLLELAILD
SdPCS       : ---FLSSLVRHSLELPLHWSVQRPNARWFANAYSKKSQANPILLELAKLD
SdLiS       : --KNLSLWIQHSLEIPLHWRTPNLEAKWMIDAYSRRPDFNPTIL-LAKLD
ClLS_AF514  : --VFVAEQAKRALELPLHWKVPMLEARWFIHIYERREDKNHLLLELAKME
SdSaS       : ----LKEQVRHALEVPLHWRLQKLEARWFIQAYEDTAEANRTLVELAKLE

                    *       320         *       340         *
SdLS        : INIVQAQFLEELKETSRWWESTGLAQELPFVRDRIVECYYWTTGVIERRE
SdEuS       : INIVQAQFLEELKETSRWWESTGLAQELPFVRDRIVECYYWTTGVVERRE
PfLS_KM015  : LNIFQAQFQQELKQDLGWWKNTCLAEKLPFVRDRLVECYFWCTGIIQPLQ
SdPS        : LNVVQAQFQDELKQDLGWWRNTSFVEKLPFARDRLVECYFWTTGIVQPRQ
SdPCS       : FNIVQATYQQELKEISRWWKETELAEKLSFARDRVVENYIWNVGLLFKPQ
SdLiS       : FNIVQATQIEELKDISRWWNNSCLSEKLPFVRDRLVESYFWAIGLFETHQ
ClLS_AF514  : FNTLQAIYQEELKEISGWWKDTGLGEKLSFARNRLVASFLWSMGIAFEPQ
SdSaS       : YNMVQAAYQQELKLLSSWYKETGLPEKLEFARHQLAVSYLWALGFIPEHH

                          360         *       380         *       400
SdLS        : HGFERIMLTKVFALIAAIDDIYDVYGTLEELQLFTDAIRRWDIE-SIDKL
SdEuS       : HGFERIMLTKINALVTTIDDIYEIYGTLEELQLFTDAILRWDIE-SIDKL
PfLS_KM015  : HENARVTIAKVNALITTLDDIYDVYGTLEELELFTEAIRRWDVS-SIDHL
SdPS        : HTNPRITVGKVNALITTIDDVYDVYGTLEELEQFTEAIRRWDIS-TIDQL
SdPCS       : YGYARIMTTKLFILITVIDDVFDVYGTLEETQLFKNATLRWDDE-AIDQL
SdLiS       : HCYERKTAAKIITLITSLDDVYDIYGTLDELEVFTLTIQRWDTE-AINRL
ClLS_AF514  : FAYCRRVLTISIALITVIDDIYDVYGTLDELEIFTDAVERWDINYALKHL
SdSaS       : LVYSREILSKIAVMITIIDDIYDVYGTLEELQLFTHTIERWDIN-SLDSL

                    *       420         *       440         *
SdLS        : PPYMKVCYLALYNFVNEMGYYTLKDKGFNSIPFLRKVWVDLVERYLIEAN
SdEuS       : PPYMKVCYLALYNFVNEMGYYTLKDKGFNSIPFLRKTWVDLVERYLIEAN
PfLS_KM015  : PNYMQLCFLALNNFVDDTAYDVMKEKDINIIPYLRKSWLDLAETYLVEAK
SdPS        : PSYMQLCFLALDNFVNDTAYDVLKKHGFNAIPYLRKSWRDLVEAYLIEAK
SdPCS       : PYYMKICYMVLDSFINEMAYHVLKEKDVLVIQDLRKTWGDLCSAYAKEAE
SdLiS       : PYYIQLFYLVIHNFVFELAH-ILKEEGVISIPYLQKPWVDLVEAYLQEAK
ClLS_AF514  : PGYMKMCFLALYNFVNEFAYYVLKQQDFDLLLSIKNAWLGLIQAYLVEAK
SdSaS       : PEYMKICFLALFNTVNEFAYHILRDQGFNIISNLRNLWAELCRAYYLEAT

                          460         *       480         *       500
SdLS        : WYHKGYKPSLEEYINNAWITVGGIPGLSHLFFRVTDSIDEEAAESVHKYH
SdEuS       : WYHKGYKPSLEEYINNAWMSIGGVPILSHLFFRLTDSIEEEASESVHEYH
PfLS_KM015  : WFYSGHKPNLEEYLNNAWISISGPVMLCHVFFRVTDSITRETVESLFKYH
SdPS        : WYHSAHKPNLEEYLNISWISIGATVILTHAFFGVTPAITKDACDALYGYH
SdPCS       : WYHTGYKPTMEEYINVAWISISAHTIITHVFYLISNPIEKEAAESIRNYH
SdLiS       : WYYGGYTPSLEEYLNNSSISIGTPTVIAQVFLTSKD--------KLYKDN
ClLS_AF514  : WYHSKYTPKLEEYLENGLVSITGPLIITISYLSGTNPIIKKELEFLESNP
SdSaS       : WFHSGYFPTTNEYLNTAWISISGPLLLFYGYFT-TNPINQKELKSLEQYP
```

```
                         *       520         *       540         *
SdLS        : EIVRASCTITRLADDMGTSLAEVKRGDVPKSVQCYMNESNASEEEARENV
SdEuS       : DIVRASCMILRLADDMGTSLDEVERGDVPKSVQCYMNENNASEEEARKHV
PfLS_KM015  : DLIRYSSTILRLADDLGTSLEEVSRGDVPKSIQCYMNDNNASEEEARRHI
SdPS        : DIVRWSAIILRLTDDLGTSLDEVSRGDVPKSIQCYMNDNNASEEEARAHV
SdPCS       : DTIRYSAMVLRLADDLGTSPHEMQRGDVPKAVECYMNETGASREEAREHV
SdLiS       : HIIHLSGMLVRLPDDLGTLVFEMKRGDVAKSIQCYMKERNASMEEAEEHV
ClLS_AF514  : DIVHWSSKIFRLQDDLGTSSDEIQRGDVPKSIQCYMHETGASEEVARQHI
SdSaS       : GIIRWPATVLRLADDLGTSSDEMKRGDVPKSIQCYMKETGCSEEDARKHI

                    560         *       580         *       600
SdLS        : QSLIEETWKTMNKEMM--DSPFSTCFVEVCANIARMAQLIYQKDSDGFGM
SdEuS       : RSLIEQTWKMMNKEMM--DSQFAIYFVEVCANLGRMAQFIYQKESDGFGM
PfLS_KM015  : RWLIAETWKKINEEVWSVDSPFCKDFIACAADMGRMAQFMYHN-GDGHGI
SdPS        : KWMIGETWKKMNEARVAKDSPFCEDFVGCAVDLGRMAQYMYHY-GDGHGI
SdPCS       : MFMIREIWMKTNGERFR-ESPFSDDFIRSAADLGRQAQYMYQH-GDGHGI
SdLiS       : RFLIREAWKEMNTAGG---CPVRDDLVETAANFGRAAQFMYLD-GDGN--
ClLS_AF514  : KDMMRQMWKKVNAYTADKDSPLTGTTTEFLLNIVRMSHFMYLH-GDGHGV
SdSaS       : KQLIDTALKRMNKEILMEN-PIKNFG-QTAMNLGRISLCMYQH-GDGFGL

                         *       620         *
SdLS        : QHSVVNKQIRSLLFEPYE----------------
SdEuS       : QHSLVNKQIRSLLFEPYE----------------
PfLS_KM015  : QNPQIHQQMTDILFEQWL----------------
SdPS        : QHPIIHQQMTDCLFHPIA----------------
SdPCS       : SNPEMEERILGLIFQPIV----------------
SdLiS       : -HSKLHQRIASLLFQPCD----------------
ClLS_AF514  : QNQETIDVGFTLLFQPIPLEDKHMAFTASPGTKG
SdSaS       : PYSETKKNIMSLLVQPFSMP--------------
```

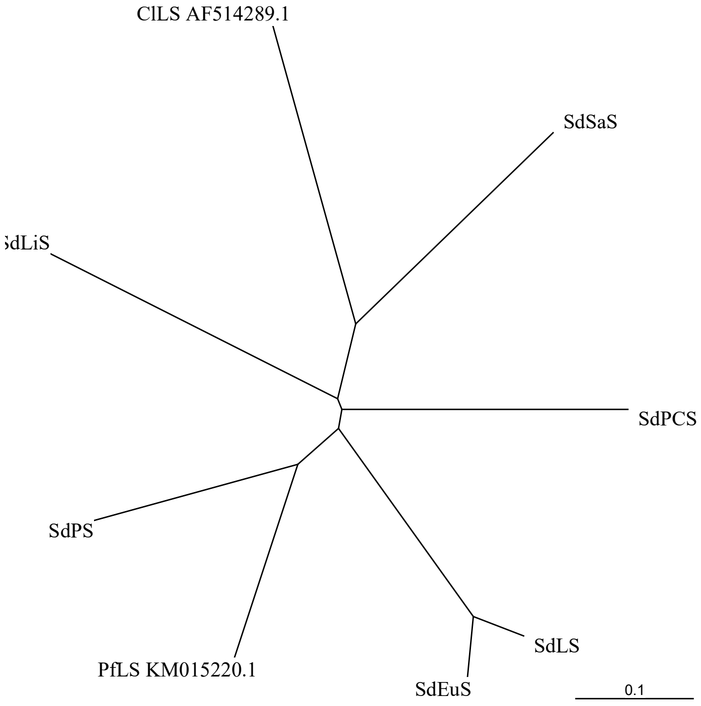

Figure S6 GC-MS TIC chromatograms of *N. benthamiana* headspace after agro-infiltration. pBin empty (empty vector control), PfLS *Perilla frutescens* limonene synthase (positive control), SdLS the newly identified limonene synthase from *Salvia dorisiana*

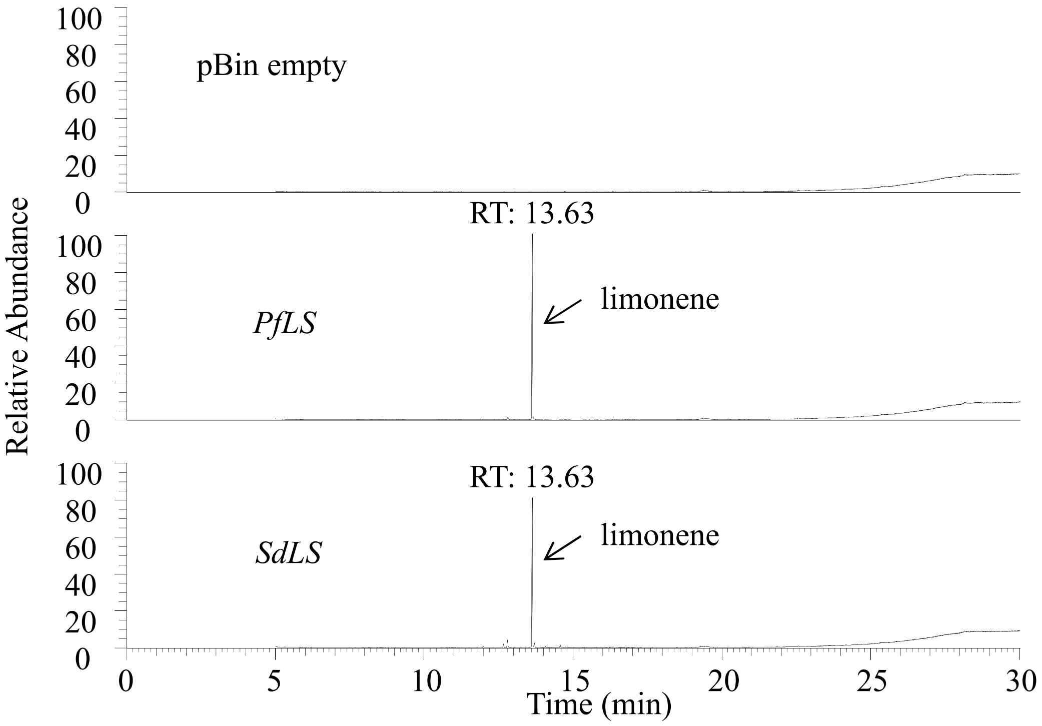

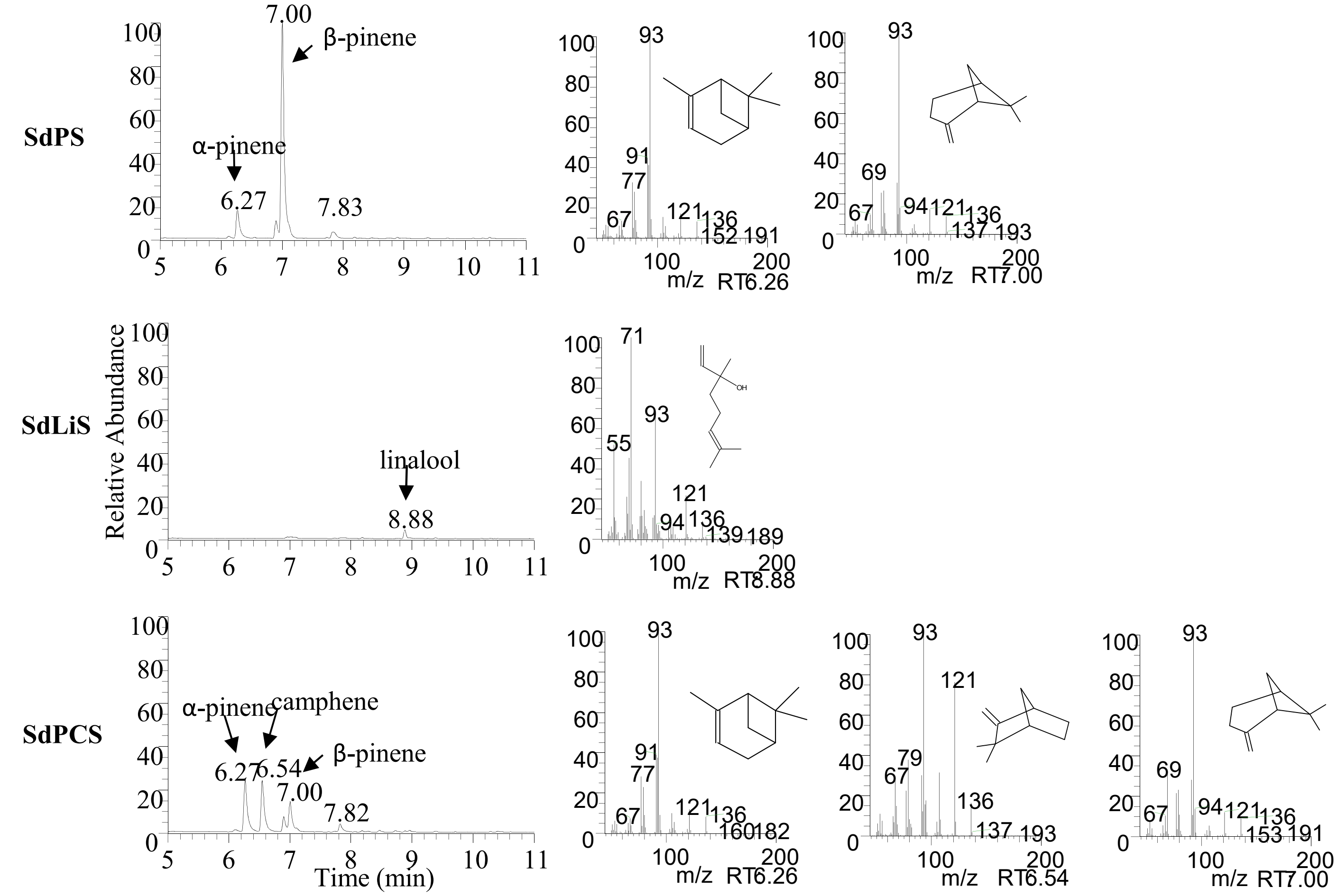

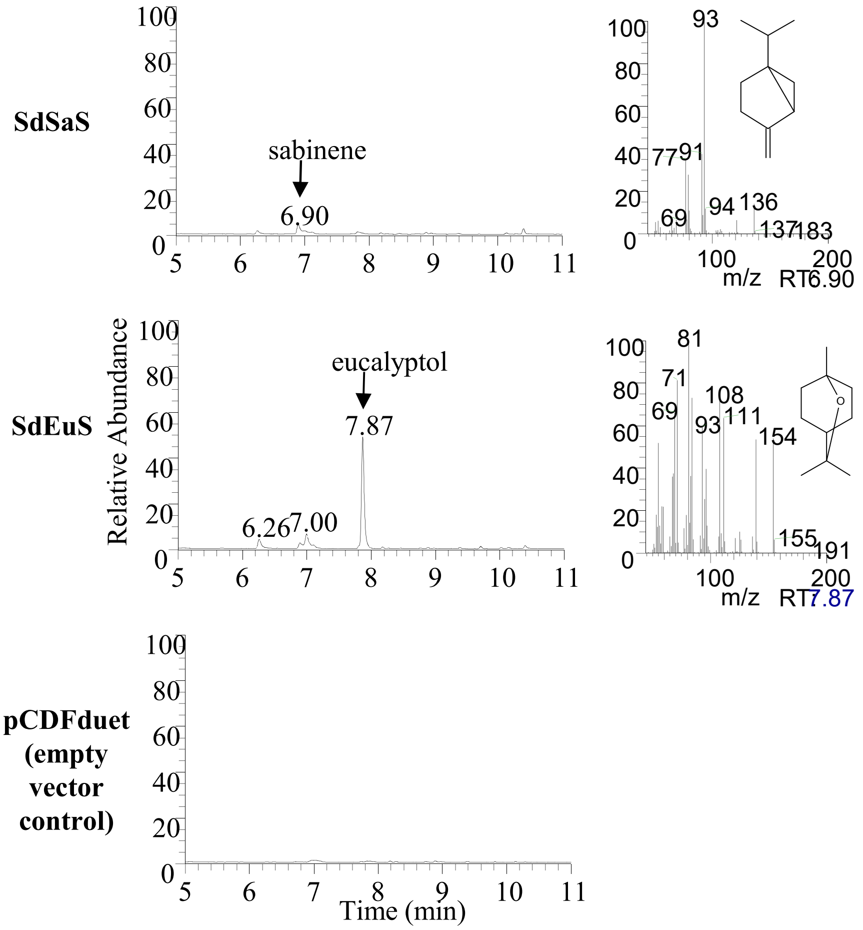

Fig. S7 In-vitro assay of other *Salvia dorisiana* monoterpene synthases, GC-MS chromatograms. SdPS β-pinene synthase, SdLiS linalool synthase, SdPCS pinene/camphene synthase, SdSaS sabinene synthase, SdEuS eucalyptol synthase, pCDFduet empty vector control. Mass spectra and structures of indicated peaks are depicted on the right of each GC-chromatogram.

Figure S8 Protein sequence alignment of P450s. SdL7H and other cloned *S. dorisiana* P450 enzymes are indicated with systematic names (Nelson, 2009), L3H *Mentha spicata* (Lucker et al., 2004), L6H *Mentha spicata* (Lupien et al., 1999), PfL367H *Perilla frutescens*, sequence based on genbank GQ120438.1, cDNA from *P. frutescens* leaves isolated, 5'-race pcr, full length gene amplified and sequenced.

```
                                    *        20         *        40         *
MsL3H_AY622319.1: -MELQISSAIIIIVATF----VASLLIKQWRKSESRQNLPPGPPKLPL
MsL6H_AAD44150.1: -MELDLLSAIIIIVATY----IVSLLINQWRKSKSQQNLPPSPPKLPV
Pf_L367H        : MYSMELELPSINILLGLLFSSYCIILLLIKQWKTTENRGKLLPSPPKLPV
SdL7H_CYP71A76_v1: ----MAALLLLISFMF--------LVLFFFKKSPSTKRLPPSPLKLPI
SdCYP71A76_v2:    --------MAALLLLISFMF--------LVLFFFKKSPSTKRLPPSPLKLPI
SdCYP71A77      : --------MAALLLILSFIF--------LVHLLLKKS-STKRLPPSPLKLPI
SdCYP76AK17     : ---------MGGFLLYIVAF----VAALAAVVLSR-RRRGELPPGPVPLPI
SdCYP76AK18     : ---------MQLLIVLSLAF----LAAWLVYSTWQGRSHGKSPPGPPRLPI
SdCYP76AH36     : ---------MDSFPFLAALF----LITAATWLISS-RRRKNLPPGPFPYPI

                         60         *        80         *       100
MsL3H_AY62  : VGHLHLLWGKLPQHAMADMAKKYGPVTHVQLGEVFSVVLSSREATKEAMK
MsL6H_AAD4  : IGHLHFLWGGLPQHVFRSIAQKYGPVAHVQLGEVYSVVLSSAEAAKQAMK
Pf_L367H    : IGHLHLMVGRLPQHVLTRAAQKYGPVMHIQLGEIFSVVVSSREATKQVMK
SdL7H_CYP7  : IGNIYLAG-SLPHRSFQSLSKRYGEVMLIHFGSKPVVVASSANAAREIMK
SdCYP71A76  : IGNIYLAG-SLPHRSFQSLSKRYGEVMLIHFGSKPVVVASSANAAREIMK
SdCYP71A77  : IGNIYLAG-SLPHRSFHSLSKRYGEVMLIQFGSKPVVVASSAKAAREIML
SdCYP76AK1  : VGNIFQIR-GDPHKAFAELAKTYGPLMSIRLGTQLAVVISSSEVAMEVLQ
SdCYP76AK1  : IGNMLQLG-RDPHKSLARLAKTYGPLMSIKLGNQLAAVVSSPELAKEVLH
SdCYP76AH3  : VGNMLQLG-SQPHETFAKLSKKYGPLMSINLGSLYTVIVSSPEMAKEIMH

                    *       120         *       140         *
MsL3H_AY62  : LLDPACADRFESIGTRIMWYDNDDIIFSPYSD-HWRQMRKICVSELLSAR
MsL6H_AAD4  : VLDPNFADRFDGIGSRTMWYDKDDIIFSPYND-HWRQMRRICVTELLSPK
Pf_L367H    : GLDPACADRADSIGTKIMWYDNKDLIFSPYNA-HWRQMRKICVSELLNAR
SdL7H_CYP7  : NQDLIFASRPRLSFIDRFFYGGRDVAFAAYGD-SWRKGRSMCVLHLFSSK
SdCYP71A76  : NQDLIFASRPRLSFIDRFFYGGRDVAFAAYGD-SWRKGRSMCVLHLFSSK
SdCYP71A77  : NQDLIFASRPRFSTADRLLYGGRDIAFAAYGD-HWRKGRSMCVLHLVSNK
SdCYP76AK1  : KHGQSFSNRSIPDAINIHGHDKVSWNTMLADSVGWKRIRKLGREKLFSHQ
SdCYP76AK1  : KQGRVFAKPFTPHAVRILGHDEISMNMLPSSSERWKKLRRVAREQLFSNP
SdCYP76AH3  : KYGQVFSGRTIAQAVEACGHDKISMGFLPVGG-EWRDMRKICKEQMFSHQ

                   160         *       180         *       200
MsL3H_AY62  : NVRSFGFIRQDEMSRLIRHLQSSA--GETVDMTERIATLTCSIICRAAFG
MsL6H_AAD4  : NVRSFGYIRQEEIERLIRLLGSSG--GAPVDVTEEVSKMSCVVVCRAAFG
Pf_L367H    : NEKSFGFIREDEMSRLVRFLRSSA--GQAVNMTEKITATTSSIICRAAFG
SdL7H_CYP7  : RVQSFRPIRDEETSLMIEKIKRSS--PSVVNLSEMFMSLTNDVVSRAVLG
SdCYP71A76  : RVQSFRPIRDEETSLMIEKIKRSS--PSVVNLSEMFMSLTNDVVSRAVLG
SdCYP71A77  : RVQSFRPIRDEETSLMTEKIKRFS--PGVVNLSKVFMDLTNDVVCRAVLG
SdCYP76AK1  : ALQQTEGQRQERLRKLAEHVRGFSERGQVMNVGEATFTTMTDLVFSTLFS
SdCYP76AK1  : ALQASQDIRQERLRKLVEYISKCSGEGRAMNVGEATFTTMTNLMFSTLFS
SdCYP76AH3  : SMEGSQDLRKLKLQQLIDYAHKCSDEGRAIDIREAAFITTLNLMSATLFS
```

```
                  *        220        *        240         *
MsL3H_AY62 : AIINDHEE---------LVELVKDSLSMASGFELADLFPSSKLINLLCWN
MsL6H_AAD4 : SVLKDQGS---------LAELVKESLALASGFELADLYPSSWLLNLLSLN
Pf_L367H   : SVVRDDEV---------LIGLVKTASGMANGFELADLFPSSKLINLLCLN
SdL7H_CYP7 : RTYGGDDDG-----EKNFNQILKKIVEILQSYNVGDFVAWLGWINRVNGV
SdCYP71A76 : RTYGGDDDG-----EKNFNQILKKIVEILQSYNVGDFVAWLGWINRVNGV
SdCYP71A77 : RKYGGGDDR-----EKPFNQILKMIVEMLQRYNVGDFVPWLGWINRVNGV
SdCYP76AK1 : IDLTDYGATDSIANK-EFKEHINSFTRYIGVPNVSDFYPILAPLDLQGVR
SdCYP76AK1 : IELVEYASTDAATPKSKFREHVNAITRYMGVPNVADFFPIFAPLDPQGMR
SdCYP76AH3 : LQATEFDS----KATMEFKEIIEGVASIVGVPNFADFFPILRPFDPQGVK

                    260        *        280        *        300
MsL3H_AY62 : KSKLWRMRRRVDTILEAIVDEHKLKKS--GEFGGEDIIDVLFRMQKDSQI
MsL6H_AAD4 : KYRLQRMRRRLDHILDGFLEEHREKKS--GEFGGEDIVDVLFRMQKGSDI
Pf_L367H   : KYRLWKMRRELDAILEGVVEEHKLKQS--GEFGGEDIVDVLIRMQKNSQL
SdL7H_CYP7 : EAQVEKIFEMTDEFMEALLREYRDKKS--SGDAVVNFADALIELQGESKD
SdCYP71A76 : EAQVEKIFEMTDEFMEALLREYRDKKS--SGDAVVNFADALIELQGESKD
SdCYP71A77 : EAQVEKIFKMTDQFMEGLLREYREKKS--SDDAVVNFADALIQLQRESKD
SdCYP76AK1 : RKIGHHLGKLLDFVEDMIQKRMRERRE-SDYQNKNDFLDTLIEIAEETE-
SdCYP76AK1 : RKLTYHLGSVLELVQGFIEQRLQARTT-SSYQKKTDFLDTLIDLSEGNE-
SdCYP76AH3 : RRADVYFGRLLGLIEGYLNERIQSRKANPNAPKKDDFLETLVDILEAND-

                  *        320        *        340         *
MsL3H_AY62 : KVPITTNAIKAFIFDTFSAGTETSSTTTLWVMAELMRNPAVMAKAQAEVR
MsL6H_AAD4 : KIPITSNCIKGFIFDTFSAGAETSSTTISWALSELMRNPAKMAKVQAEVR
Pf_L367H   : QFPITTDTIKGFIFDTFAAGTETSSTTTVWAMAELMKNPRVMANVQAEVR
SdL7H_CYP7 : SDPVEDDVIKALILDTFAAGTDTTFTALEWTMAELIRNPRTMKLLQKEVR
SdCYP71A76 : SDPVEDDVIKALILDTFAAGTDTTFTALEWTMAELIRNPRTMKLLQKEVR
SdCYP71A77 : SDPVEDDVIKALILDMFGGGTDTTFTALEWTMAELIRNPRTMKLLQKEVR
SdCYP76AK1 : -YDLTIKEIRHFCVDILIAGSDTSAATTEWAMVELLLHPDKMAKLKAELK
SdCYP76AK1 : -YDLSVKEIKHMFVDIIIAGSDTSAATSEWAMVELLLHPDKMAKLKAELK
SdCYP76AH3 : -YKLKTEHLTHLMLDLFVGGSETSTTEIEWIMEELMSSPDKMAKVKAELK

                    360        *        380        *        400
MsL3H_AY62 : AALKGKTSVDVD--DVQELKYMKSVVKETMRMHPPIPLIP-RSCREECEV
MsL6H_AAD4 : EALKGKTVVDLS--EVQELKYLRSVLKETLRLHPPFPLIP-RQSREECEV
Pf_L367H   : EGLKGKKSVDAS--DVQQLKYLKSVVKETLRLHPPFPLIP-RKCREDIEV
SdL7H_CYP7 : EVARNKNGIDINEDDLEKMPYLKAVSKESLRLHPPLPLALPRELNQDTNL
SdCYP71A76 : EVARNKNGIDINEDDLEKMPYLKAVSKESLRLHPPLPLALPRELNQDTNL
SdCYP71A77 : EVARNKNGIDINEDDLDKMPYLKAVSKESLRLHPPLPLAVPRELTQDTNL
SdCYP76AK1 : SVLGDKSTVEGS--DISKLPYLQATINEVFRFHPVAPLLGPREAAEDTQI
SdCYP76AK1 : SVLGSKKMVEEA--DISRLPYLRATVKEVFRYHPAAPMLAPHAAEEQTEV
SdCYP76AH3 : SVMGEQKVVDER--QMPNLPYLQAVVKESMRLHPPGPLLLPRKAESDQVV
```

```
                          *         420         *         440         *
MsL3H_AY62 : NGYKIPNKARIMINVWSMGRNPLYWEKPETFWPERFDQVSRDFMGSDFEF
MsL6H_AAD4 : NGYTIPAKTRIFINVWAIGRDPQYWEDPDTFRPERFDEVSRDFMGNDFEF
Pf_L367H   : EGYSIPSNSRIVINVWSLGRDPLYWEEPEIFWPERFDHISTDYVGNNFEF
SdL7H_CYP7 : LGYDIPRGALVLVNCWAISRDPLLWENPNEFRPERFFDSSIDYKGLHFEM
SdCYP71A76 : LGYDIPRGALVLVNCWAISRDPLLWENPNEFRPERFFDSSIDYKGLHFEM
SdCYP71A77 : LGYDIPRGALVLVNCWAISRDPLLWENPNEFRPERFFNTSIDYKGLHFEM
SdCYP76AK1 : NGYTIPKNVKVIVNFWAITRDRSIWKNPESFEPERFLGTDINFEGQHFEL
SdCYP76AK1 : NGYIIAKNTKIFINFWAITRDPSIWKNPESFEPERFLDSEIDFGGQHYEL
SdCYP76AH3 : NGYLIPKGSQVLINAWAMGRDETIWKNPNSFEPERFLDQKIDFKGQDYEL

                      460         *         480         *         500
MsL3H_AY62 : IPFGAGRRICPGLNFGLANVEVPLAQLLYHFDWKLAE-GMKPSDMDMSEA
MsL6H_AAD4 : IPFGAGRRICPGLHFGLANVEIPLAQLLYHFDWKLPQ-GMTDADLDMTET
Pf_L367H   : IPFGGGRRICPGLNLGVANVEVPLAQLLYHFDWKLGEPGMSPVHMDMTVA
SdL7H_CYP7 : VPFGAGRRGCPGIAFAMSMYELAVSRLVKEFDFGLPN-GVREEDLDMTEA
SdCYP71A76 : VPFGAGRRGCPGIAFAMSMYELAVSRLVKEFDFGLPN-GVREEDLDMTEA
SdCYP71A77 : IPFGAGRRGCPGIGFAMSLYELALSRLVNEFDFGMPN-GGREEDLDMTEA
SdCYP76AK1 : IPFGSGRRICPGMPLASRMLFCMVATLCHNFDWELEG-GVESKLREREDV
SdCYP76AK1 : IPFSSGRRICPGMPLAGRMLHCMVGTMCHNFDWELEK-GAESKQLQREAV
SdCYP76AH3 : IPFGSGRRVCPGMPLANRILHTVTATLVHNFEWKLER-PDASDAERQGVL

                        *         520
MsL3H_AY62 : EGLTGIRKNNILLVPTLYKSP---
MsL6H_AAD4 : PGLSGPKKKNVCLVPTLYKSP---
Pf_L367H   : KGLSGPRKTPLFLVPSIYIPTQPN
SdL7H_CYP7 : PGFVVHKKSPLLVVTTPRAY----
SdCYP71A76 : PGFVVHKKSPLLVVATPRASL---
SdCYP71A77 : PGFAVHKKSPLLVVATPRASL---
SdCYP76AK1 : FGLALQKKFPLRAKPIKV------
SdCYP76AK1 : FGLALQKKIPLRAIPIKVE-----
SdCYP76AH3 : FGFAVRRAVPLKVIPFKK------
```

Fig. S9 Characterization of *SdL7H* in yeast. Mass spectra of the peaks shown in Fig. 4. a. POH peak from yeast strain ScPftLS + SdL7H at RT 11.3 min, b. POH standard at RT 11.3 min.

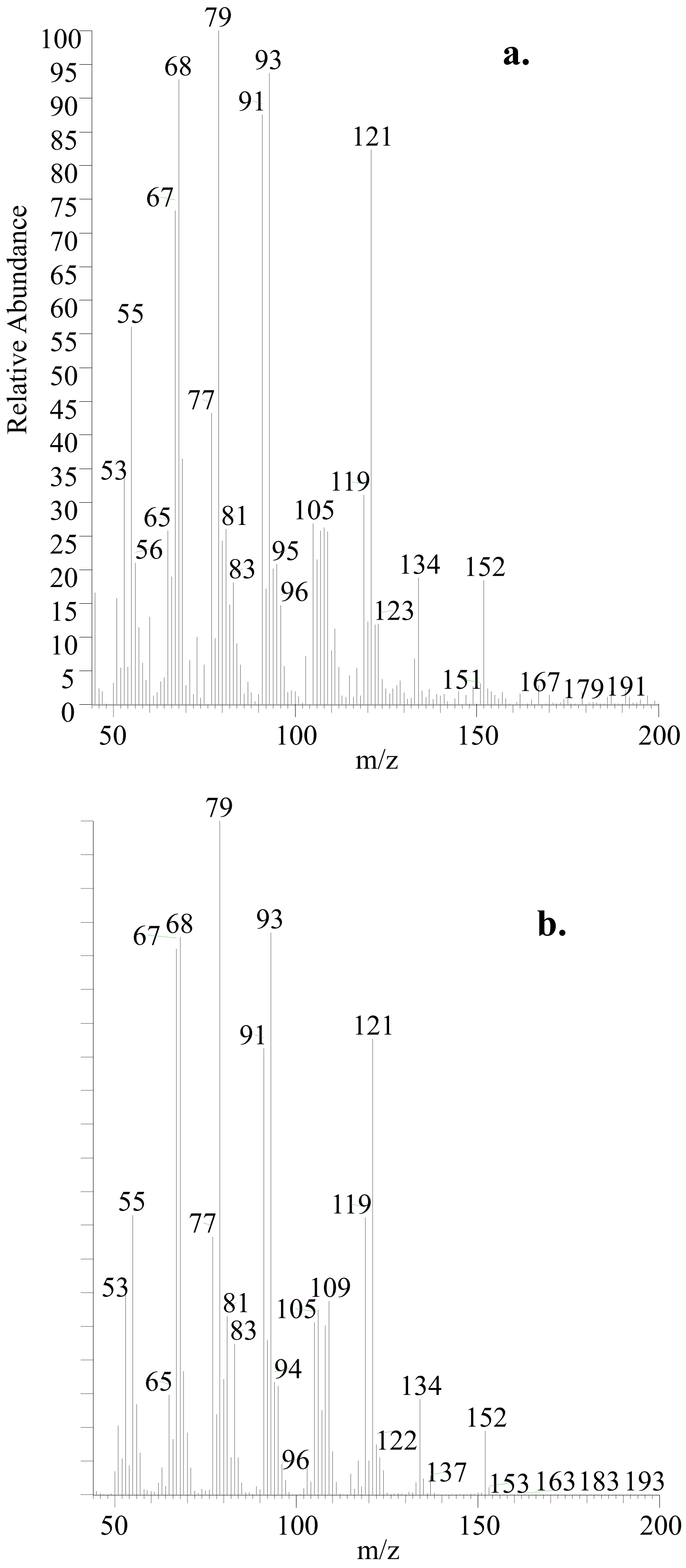

Fig. S10 a. GC-MS chromatograms of *N. Benthamiana* leave headspace after agro-infiltration of constructs, b. Mass spectrum of perillyl alcohol peak in SdLS+SdL7H at RT 18.04 +c Full ms [35.00-400.00], c. Mass spectrum of perillyl alcohol reference compound. NL 5.17E7, m/z = 92.5-93.5+120.5-121.5+151.5-152.5 MS. d. LC-MS chromatograms of *N. benthamiana* agro-infiltrated leaves. m/z 355.1760-355.1770 (perillyl alcohol marker POH-M1 at RT 41.93) FTMS - c ESI Full ms [95.00-1300.00] MS NL: 100%= 4.38E6 e. GC-MS peak area of limonene peak in *N. benthamiana* leaf headspace after agro-infiltrations. *SdLS Salvia dorisiana* limonene synthase, *SdL7H S. dorisiana* P450 limonene-7-hydrogenase, *MsL3H Mentha spicata* limonene-3-dehydrogenase (P450, isopiperitenol synthase, control)

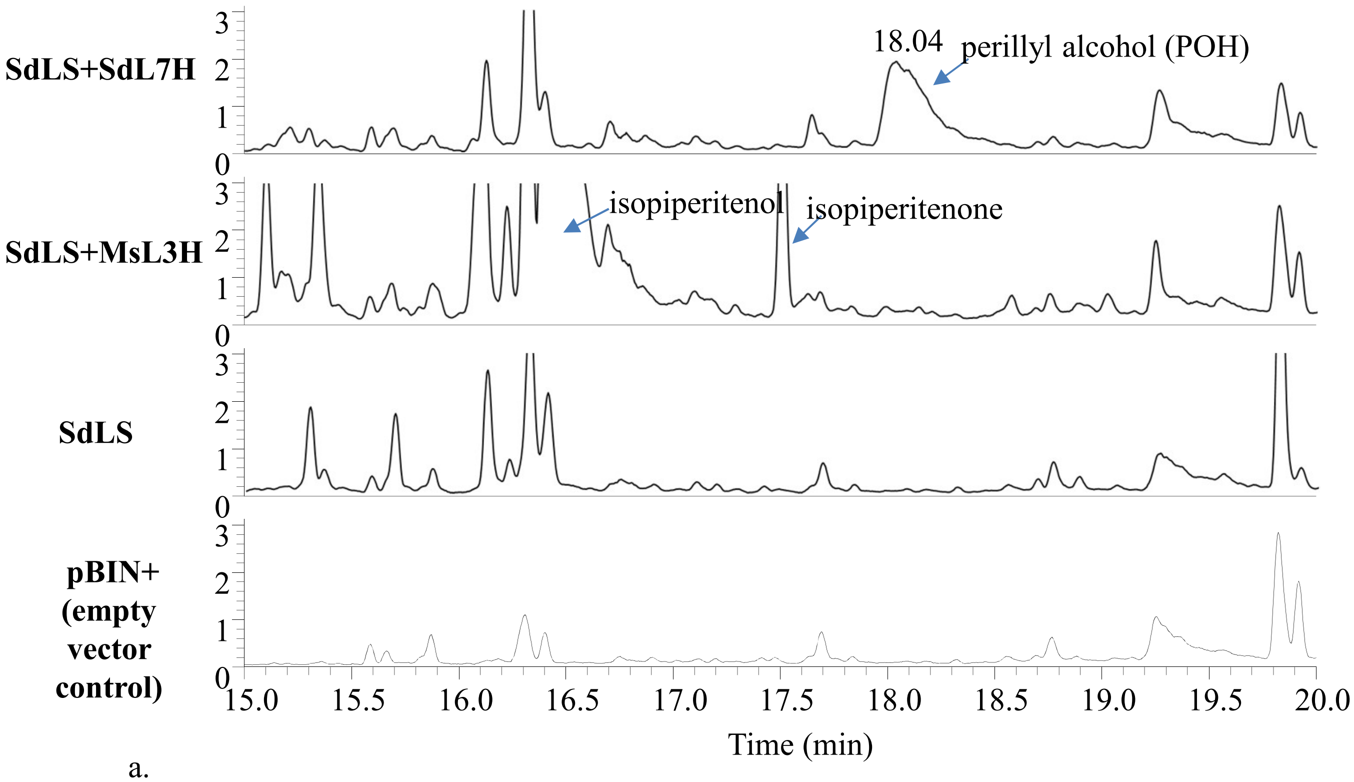

a.

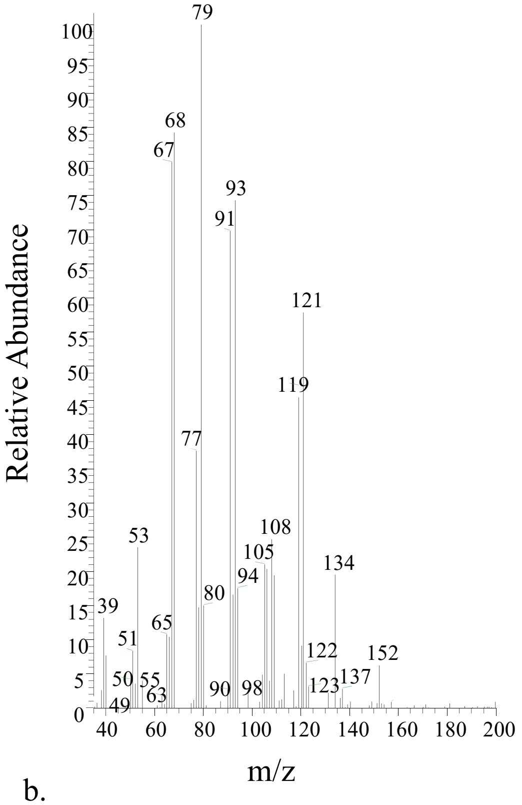

b.

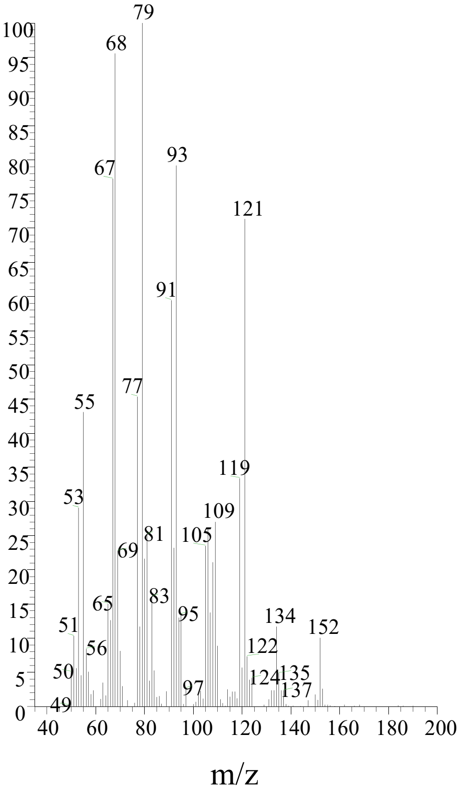

c.

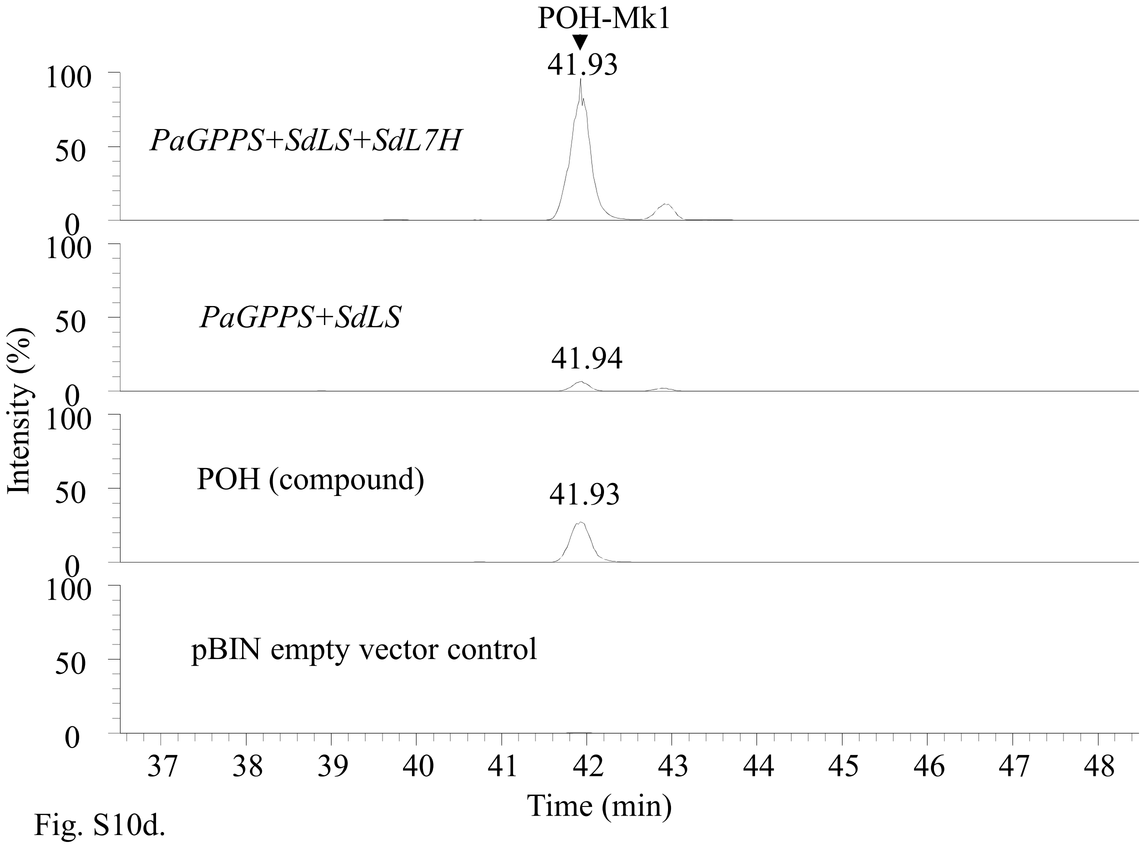

Fig. S10d.

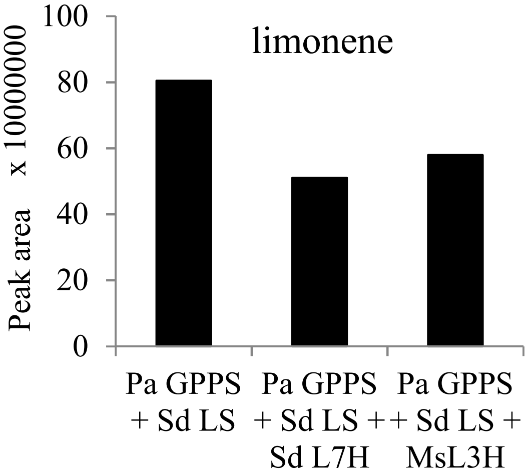

Fig. S10e.

Figure S11 Alcohol dehydrogenases (ADH) protein sequence alignment and phylogenetic tree. SdADH characterized *Salvia dorisiana* perillyl alcohol dehydrogenase, Cr8HGO *Catharanthus roseus* 8-hydroxygeraniol oxidoreductase (Miettinen et al. 2014), AaAdh2 *Artemisia annua* ADH2 monoterpene alcohol dehydrogenase Genbank No. GU253890.1 (Polichuk et al 2010), PfAKR and PfGeDH *Perilla frutescens* perilla alcohol dehydrogenases Genbank No. AFV99148 and AFY63472 (Matsumoto and Ito 2014), Sdclust... other *Salvia dorisiana* cloned ADHs that did not convert perillyl alcohol to perillyl aldehyde.

```
                       *        20         *        40         *
AaAdh2      : M-------------------ASLTPKARLENKV-----------AIVTGG
Sdclust305  : M--------WLFMRNGPSGFSSASTAEEVTNGIDAS-----GLTAIVTGA
Sdclust316  : M-------------------------ADAVDQYPAP-----RRYAIVSGA
PfAKR       : M-------------------------ATAAMTVAVP-----RIKLGVDGL
SdADH       : M------ADNTITCKAAVAWNQSEPLKVEEVEVDPPNSTQVRIKMVYASM
Cr8HGO      : MTKTNSPAPSVITCKAAVVWKSGEPPKVEEIQVDPPKASEVRIKMICASL
PfGeDH      : MAKTPETEHPVKAFGWAARDNSGTLSPFNFSRRDTGER-DVQFKVLYCGV
Sdclust424  : M--------------------------------------ENQGKRVLLTS
Sdclust351  : MAEKILIIGGTGHIGKFLVEASAKSGHPTFALMRESSLSDPPKAQIVEGW

                      60         *        80         *       100
AaAdh2      : A-RGIGECIVRLFVKHG--------AKVVIADVNDD--------------
Sdclust305  : S-SGIGLETARVLAKRG--------VNVVMGIRNMG--------------
Sdclust316  : N-RGIGFEICRQLASKG--------MVVILAARDEK--------------
PfAKR       : EVSKIGLGCVGMSETYGPPRPEPEMVQLIHHAVDSGVTFFDTSDFYGPHT
SdADH       : CHTDI-TCWNGSLFPAFPRILGHEGAGVI-ESVGEDVKNLKVGDSV-MLF
Cr8HGO      : CHTDF-LACNGLPVPLFPRIPGHEGVGMI-ESVGENVTNLKEGDIV-MPL
PfGeDH      : CHSDLHMVKNEWGVTHYPVVPGHEIVGVV-TEVGSKVEKVKAGDKVGVGV
Sdclust424  : NGDQISLSIARTLARRTCRL-------VIMGNEGQ---------------
Sdclust351  : NKSGVTI-LTGDLNDHASLVKAIKHVDIVISTVG--------------FM

                       *       120         *       140         *
AaAdh2      : ----LGKLLCQDL-----GSKFACFVHCDVTIESDIENL----INTTIAK
Sdclust305  : ----VGEKVKQGIIKENPAAKIDA-IQLDLSSMQSVRNF----ASHFKSS
Sdclust316  : ----RGIEARERLKDFNNV----VFHPLDVADAASVAAV----AHFVRQE
PfAKR       : NEILLGRALKGMREKVQIATKFGARLKGDVVEICGHPAY----VR--EAC
SdADH       : YVGECGECAYCASGKTNVCLRYPPTISD---DGTSRIYAKGQRIHRMFSS
Cr8HGO      : YLGECGECLNCKSGRTNLCHKYPLGFSGLLLDGTSRMSIGEQKVYHHFSC
PfGeDH      : IVGSCRQCDQCSNDLENYCSKQILTYGAPYIDGT-------------ITR
Sdclust424  : --------LRTAAEKIKGSLNGAVFVEVVGLDMED-------------ER
Sdclust351  : QIADQHKILAAIKEAGNVKRFLPSEFGNDVDQSHP-----ENPLSQNFQQ

                     160         *       180         *       200
AaAdh2      : HGQLDIMVNNAGTV------------------DEPKISILDNEKS-----
Sdclust305  : GLPLNILINNAGIM------------------VPPFTLSPDNIEL-----
Sdclust316  : FGRLDILVNNAASIGLLQIDGDVLVLHEHLLADAASVSSGELQELVHPKA
PfAKR       : EASLKRLETNYIDI---------YYIHRIDTRVPIEITMGELKKLVEEGK
SdADH       : STWAEYAVVESNYV----------------VKVDPRIPL-SHACLLTCG-
Cr8HGO      : STWSEYIVIEAAYA----------------VKVDPRVSL-PHASFLCCG-
PfGeDH      : GGYSNIMVADEHFI----------------IRWPENIPLDAGAPLLCAG-
Sdclust424  : EGAFDDAVEKARRILGGLDALVNCYSYEGKMQDPLSIAEDEFKKI-----
Sdclust351  : KVQLRRAIEAQGI-------------------------------------
```

```
                      *        220          *        240          *
AaAdh2     : ---------DFDRVVSINLAGVFLGT-------KHAARVMIPKCS---GS
Sdclust305 : ---------QF----ATNYLGHFLLTHLLLDTMKKTARDT--KRE---GR
Sdclust316 : NGTLIEAFEHAEECINTNYYGLKRVTEALIPLLKLSESPTIVNVSSTLGS
PfAKR      : IKHVGLSEACPSTIRRAHAVHPLAAVELEWSLWSRDSEDEIIPTCRALGI
SdADH      : --------------FTTGYGGVWK------------------ELKAGKGS
Cr8HGO     : --------------FTTGFGATWR------------------DVNVVKGS
PfGeDH     : --------------ITTYSPLRYY------------------GLDK-PGL
Sdclust424 : --------------MRTNFMAAWYLLKAVGKVMRD---------QKSGGS
Sdclust351 : ----------PYTYISCNLFAGYSLSNLLQ-----------VGATAPPRD

                    260          *        280          *        300
AaAdh2     : IITTAS------IC---SVTGGVA----SHA-----YTSSK---------
Sdclust305 : IVNLSS------IAHRNSYREGIRFDKINDE-----KSYSK---------
Sdclust316 : LVLQPNE----WVKGVLSSEDGLKEERIDEVVQEYLKNYKEGSLRENKWP
PfAKR      : GIVAYSPLGRGFLAAGPSFVENLSESDFRKRFPRFQPENIEQNKKIYERL
SdADH      : SVAVIGLGAVGLGAVKASQILGATRIFGIDVN-DMKRDIAR---------
Cr8HGO     : TVAVLGLGAVGLGAVQGAKSQGASRIIGLDIN-DKKREKGE---------
PfGeDH     : SVGVNGLGGLGHVAVKFAKAFG-TKVTVISTSLGKKKEAIE---------
Sdclust424 : IVFLTSVIGAERGLYPGAAAYGSCLAGVQQLVRTSALEIGKHQ-------
Sdclust351 : KVVIPG--------------DGNVKAVFNEEH------------------

                      *        320          *        340          *
AaAdh2     : --HGVVGIAKNA----AAE-LGKY------NIRVNCVSPYFVPTKIAFKF
Sdclust305 : --FSAYGQSKLANILHANE-LARRIKEEGADITANSVHPGVITTNI-FRH
Sdclust316 : LHMSAYKVSKAA----VNA-YTRLMAKKHDTFYINSVCPGYTRTELT-RN
PfAKR      : CEMAARRECSPAQLALAWV-LAR----------GDDVCPIPGTTKID---
SdADH      : ----AFGVTDFVNPKHSNKPISQLILEATGGLGVDYC---VECTGVASLL
Cr8HGO     : ----AFGMTEFINPKGSNKSISELINEATGGLGLDYV---YECTGVPALL
PfGeDH     : ----HLGVDEFLV---SSDP--QQMQAAVGTL--DGI---IDTVSAEHPL
Sdclust424 : -----IRVNGIARGLHLEDEFPVSVGKERAERLVNDANPLRRWLDVE---
Sdclust351 : -DVGTYTIKAAVDPRTLNKIVYFRL--------PHNIYSFNELVAIWEKK

                    360          *        380          *        400
AaAdh2     : LNMDE-TSSFYSNLQGKTLGPQDIANATLFLASDESGYVSGHNLVVDGGY
Sdclust305 : LGIFEGLVSSVGRLVLKNV--QQGASTTCYVA------------------
Sdclust316 : FGLLTDAEGAVAPVKLA-LAPEGGPSGSIFLRAE----------------
PfAKR      : -NLNQNMEAFLLELT-----PEEKADLESYASPD----------------
SdADH      : NEAIASTKMGVGETVLISAPPEEKGELNYIPMILGR-TIKGTTF----GG
Cr8HGO     : NEAIESSKVGLGTAVLIGAGLETSGEIKFIPLLCGR-TVKGSIY----GG
PfGeDH     : VPLMSLLKPH-GKLIVVGAP-EKPLQLHAFSLIQGRKTVAGSAI----GG
Sdclust424 : ---------------------NDLASTVIYLISDGARYMTGTTIFVDGGQ
Sdclust351 : INK-TLEKIYVPEQELLKQIQESPIPVNIILCINHCIFVKG---------
```

```
                   *        420         *        440         *
AaAdh2      : SVLNPAFGLFSWKP*TLSISPQCSCVLYVRFLE*GHILLVMIVKTTL*HF
Sdclust305  : ------------------LHPQLKGVSGQYFSDNN--LDKADAKATDKDL
Sdclust316  : --------------------------------------------------
PfAKR       : -MVKGERHAFMSQTWINSETPQLSNWKLENHIDDG---------------
SdADH       : VKIHSDVPKIVEKCINKEI--------------NLGDLITHEVS---LAD
Cr8HGO      : VRPKSDLPTLIEKCINKEI--------------PMDELMTHEVS---LSE
PfGeDH      : MK---ETQEMIDFAAKKNIIPDVEVIPIDYINTAMDRLLKSDVKYRFVID
Sdclust424  : SLVRPRMKSYM*--------------------------------------
Sdclust351  : ------------------------------------DQMNFEIDPKIGVE
```

```
                       460         *
AaAdh2      : ITS*FIYVY--------LIIIV
Sdclust305  : AAKLWDYTMD-------LINK*
Sdclust316  : VLSLFG-------------TN*
PfAKR       : --------------------V*
SdADH       : INKGFLEYMKQPKCVKVIIKF*
Cr8HGO      : INKGF-EYLKHPDCVKVVIKF-
PfGeDH      : VNKSFNAH*-------------
Sdclust424  : ----------------------
Sdclust351  : VSQLYPDVKYTTVDEYLNQFV*
```

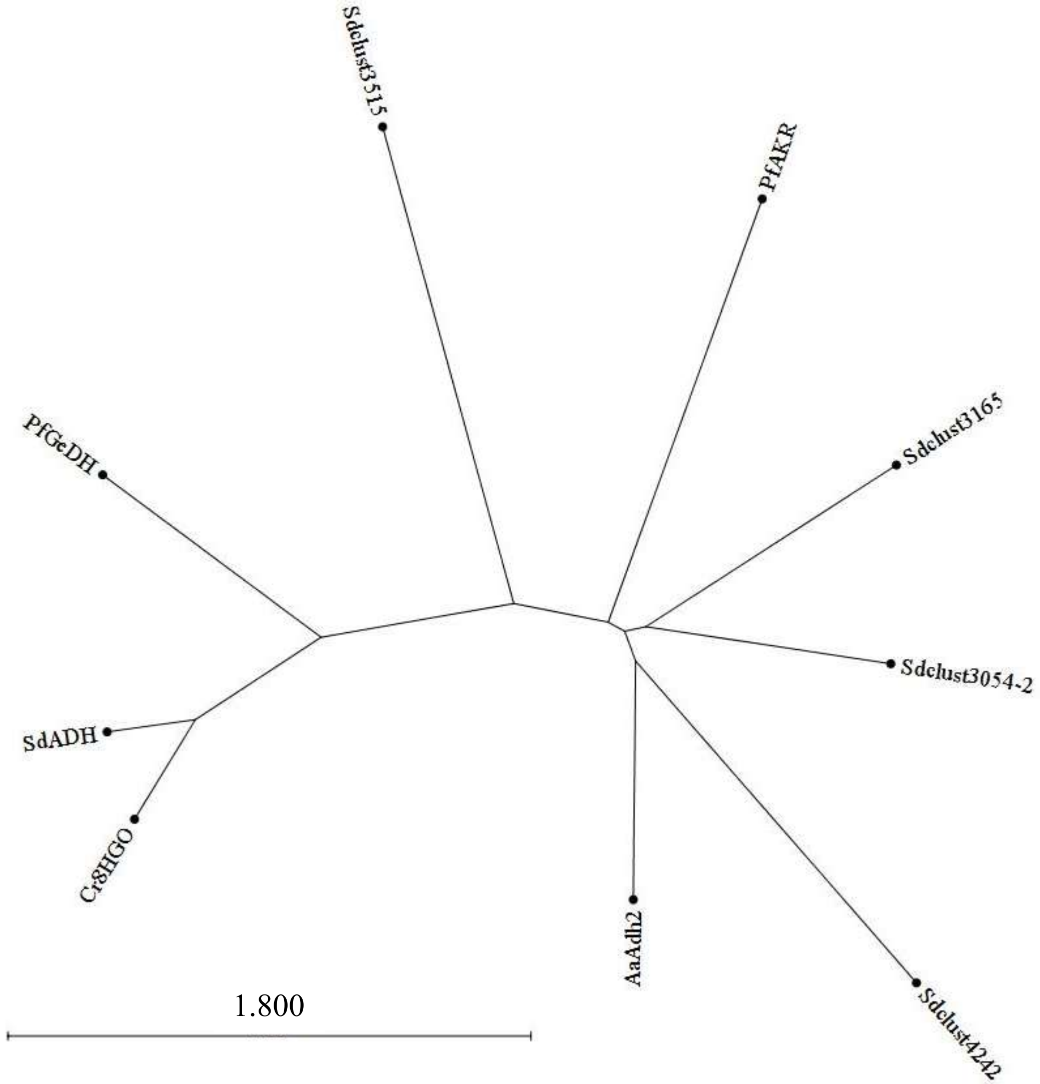

Figure S12 Characterization of *SdPOHDH* activity. a. LC-MS chromatograms of N. benthamiana leaves agro-infiltrated with *SdPOHDH* and perillyl alcohol. m/z range 355.1760-355.1770 + 458.1960-458.1970 (perillyl aldehyde marker PAldH-M1 at RT 28.11; perillyl alcohol marker POH-M1 at RT 41.93) FTMS - c ESI Full ms [95.00-1300.00] MS NL: 100%= 1.20E6 b. Quantification of peak areas of POH and PAldH markers POH-M1 and PAldH-M1 from chromatograms. POH perillyl alcohol, PAldH perillyl aldehyde, pBIN empty vector control, *SdPOHDH Salvia dorisiana* alcohol dehydrogenase.

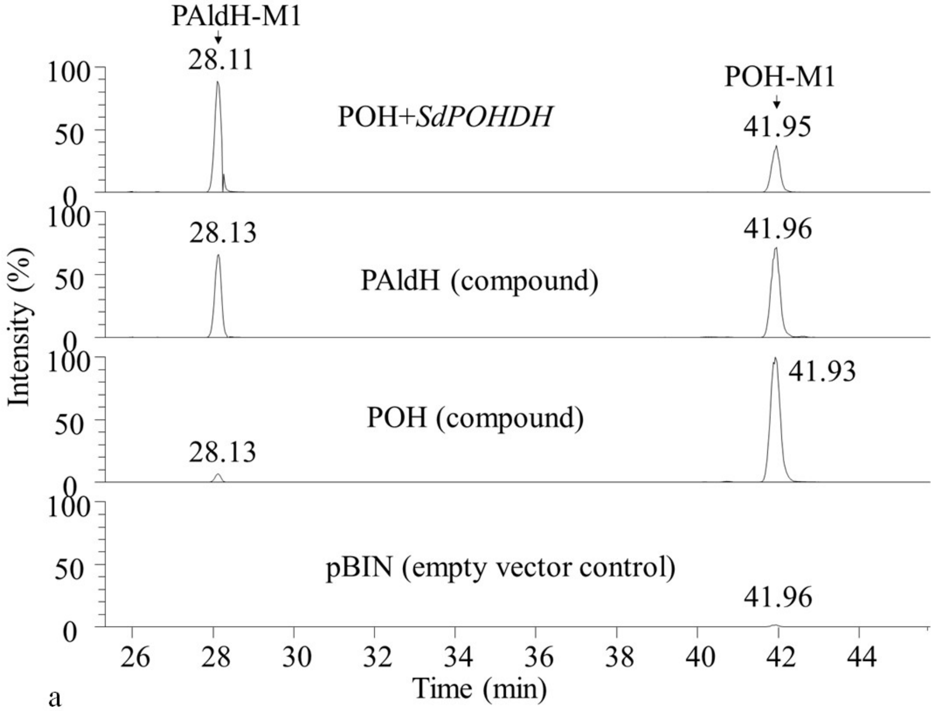

a

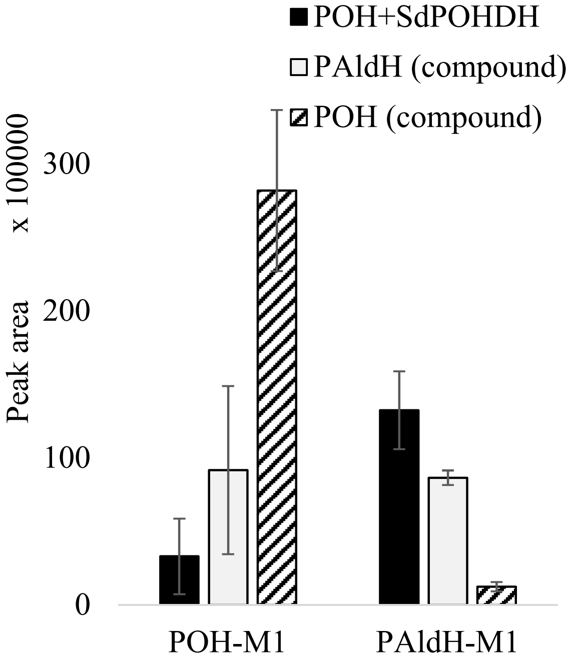

b

Figure S13 O-methyltransferases protein sequence phylogenetic tree of *Salvia dorisiana* methyltransferase candidates. SdPAOMT characterized *Salvia dorisiana* perillic acid methyltransferase (indicated with an arrow), SdOMT3,4,9,18,19 are alternative candidates from *S. dorisiana*, CrLAMT *Catharanthus roseus* loganic acid methyltransferase, ObCCMT *Ocimum basilicum* cinnamate carboxyl methyltransferase, SfSAMT *Stephanotis floribunda* salicylic acid carboxyl methyltransferase (Pott et al., 2002), AtIAMT *Arabidopsis thaliana* indole-3-acetic acid carboxyl methyltransferase (Zubieta et al., 2003), AtFAMT *Arabidopsis thaliana* Farnesoic acid methyl transferase (Yang et al., 2006), PhBSMT *Petunia hybrida* benzoic acid/salicylic acid carboxyl methyltransferase (Underwood et al., 2005), CbSAMT *Clarkia breweri* salicylic acid methyl transferase (Ross et al., 1999), CaMXMT1 *Coffea arabica* 7-methylxanthine methyltransferase (Ogawa et al., 2001), NsBSMT *Nicotiana suaveolens* benzoic acid/salicylic acid carboxyl methyltransferase (Roeder et al., 2009), AmSAMT *Antirrhinum majus* salicylic acid carboxyl methyltransferase (Negre et al., 2002), AtJMT *Arabidopsis thaliana* jasmonic acid carboxyl methyltransferase (Seo et al., 2001), AmBAMT *Antirrhinum majus* benzoic acid carboxyl methyltransferase (Dudareva et al., 2000), AtBSMT *Arabidopsis thaliana* benzoic acid/salicylic acid carboxyl methyltransferase (Chen et al., 2003), AlBSMT *Arabidopsis lyrata* benzoic acid/salicylic acid carboxyl methyltransferase (Chen et al., 2003), CsTCS1 *Camellia sinensis* caffeine synthase (Kato et al., 2000). Reference methyltransferase sequences were adapted from (Kapteyn et al., 2007).

Fig. S13.

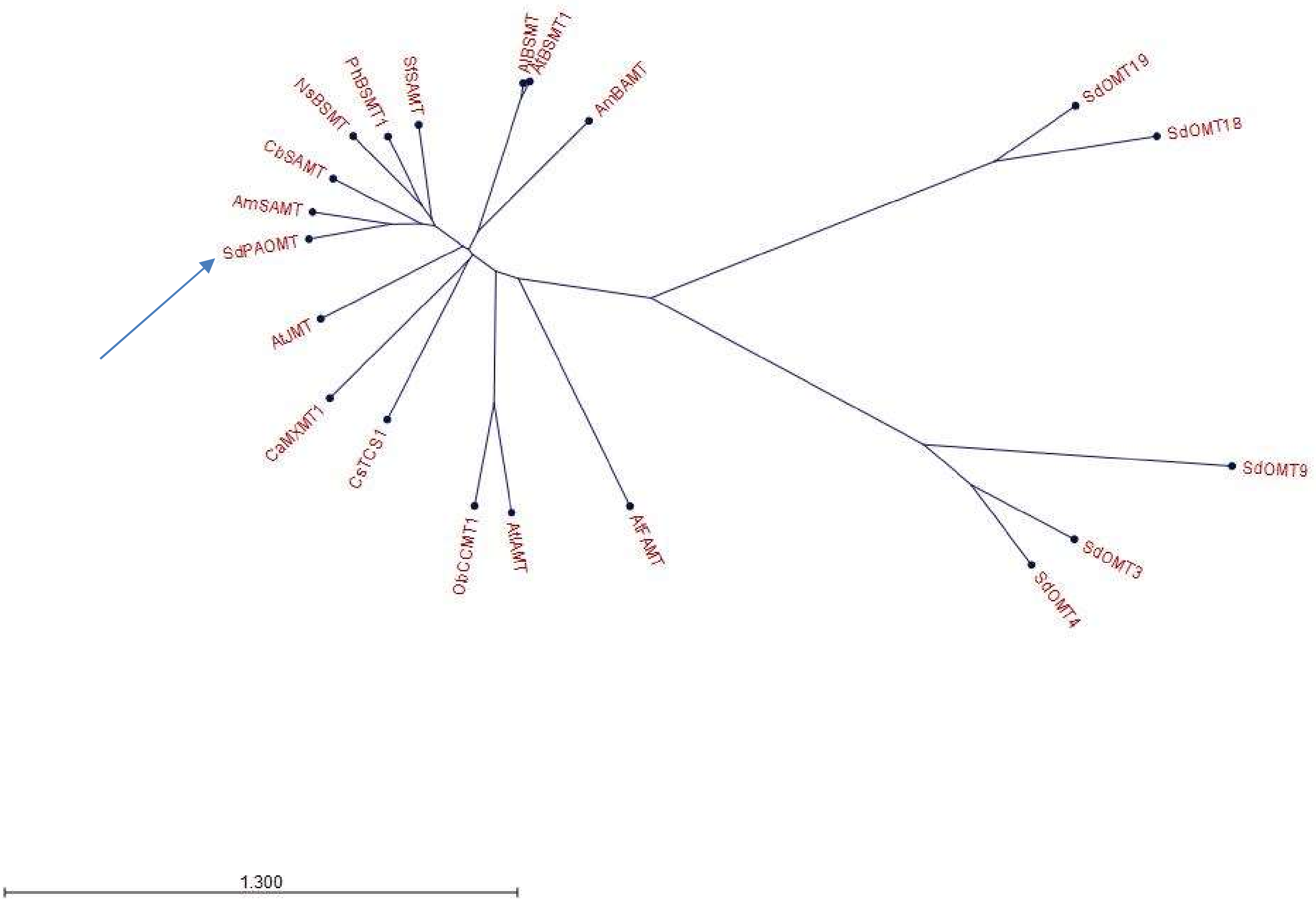
AtBSMT
AtBSMT1
PhBSMT1
SfSAMT
NsBSMT
AmBAMT
CbSAMT
AmSAMT
SdPAOMT
AtJMT
CaMXMT1
CsTCS1
ObCCMT1
AtIAMT
AtFAMT
SdOMT19
SdOMT18
SdOMT9
SdOMT3
SdOMT4
1.300

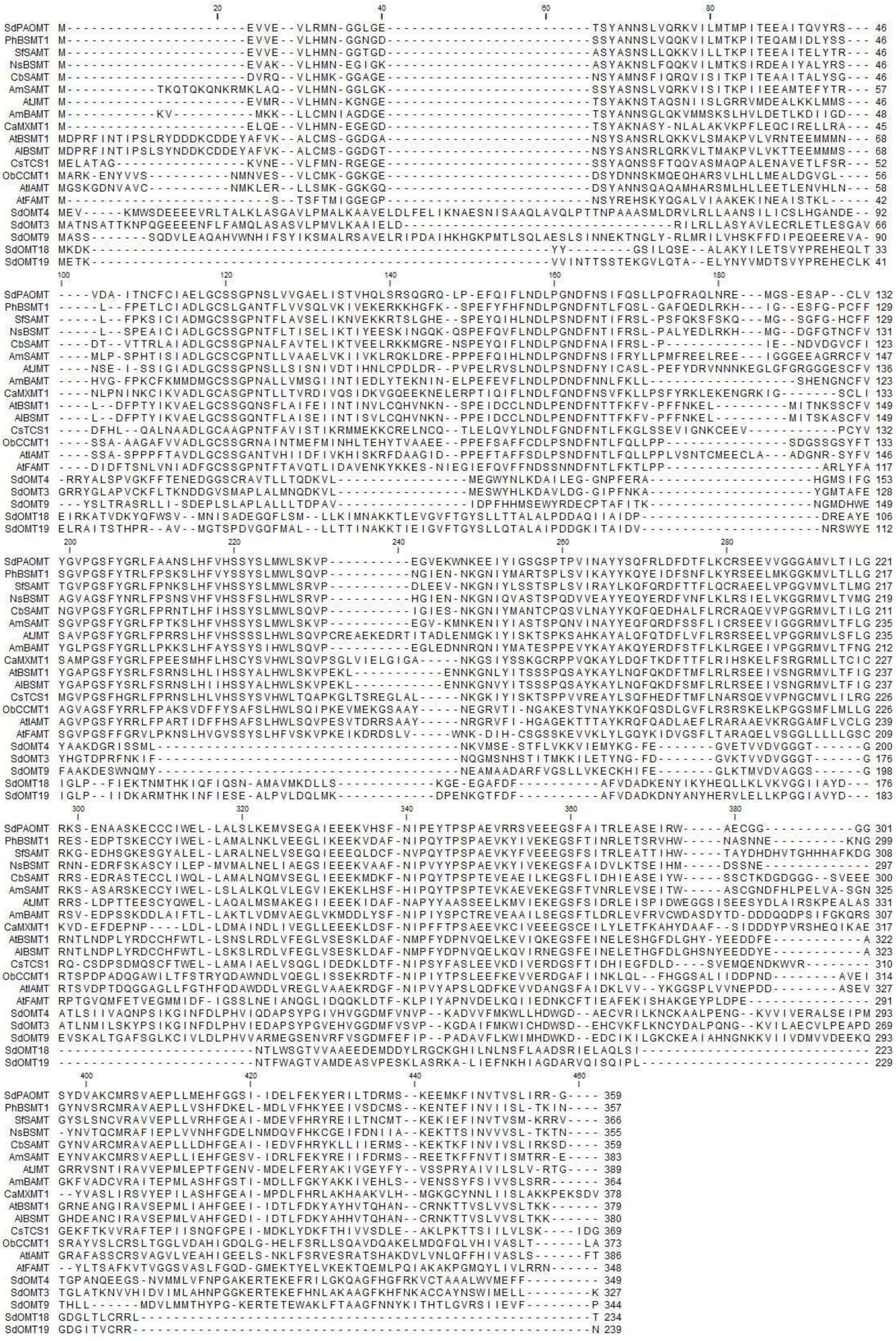

Fig. S14. Agro-infiltration of Salvia dorisiana methyltranferase candidates. Leaves of N. benthamiana were infliltrated with perillic acid and Agrobacterium cultures expressing several OMT candidates, or empty vector control. a. GC-MS chromatograms of leaf headspace; b. peak area of methylperillate m/z 180.1 extracted, quantified relative to pBIN peak empty vector control. Error bars indicate standard deviation. c. Mass spectrum of SdPAOMT product, +c Full ms [35.00-400.00], d. Mass spectrum of methylperillate reference compound, +c Full ms [35.00-500.00]

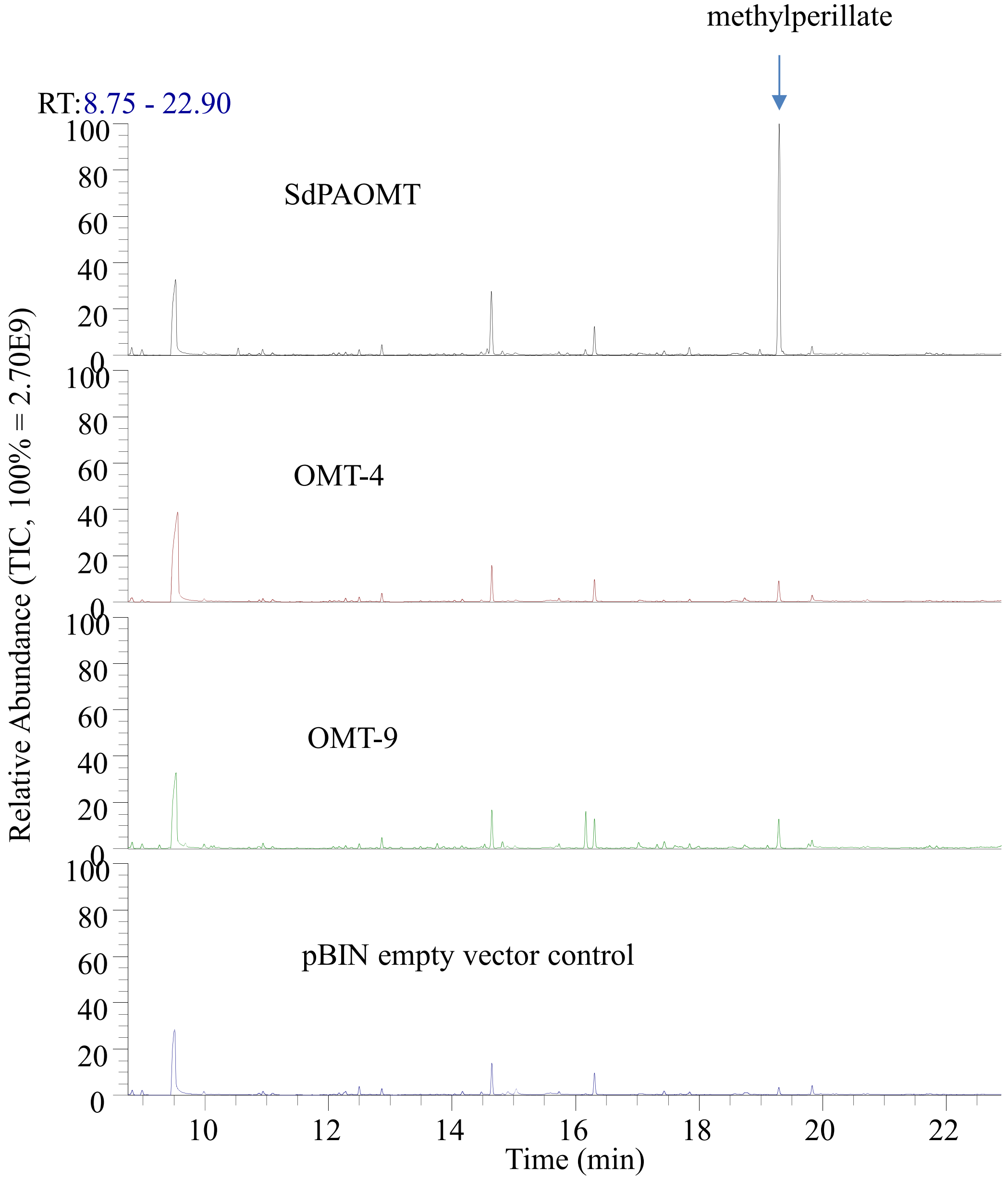

a

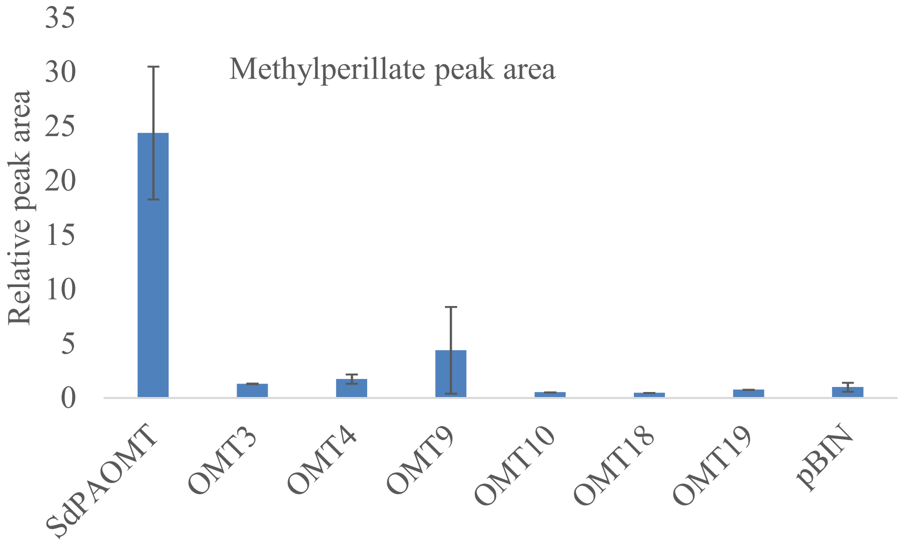

Figure S14b

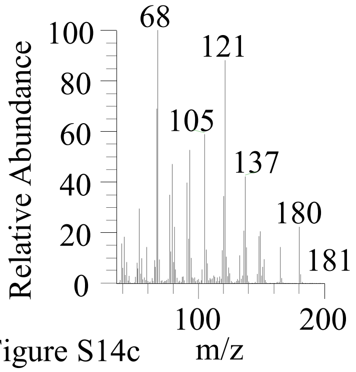

Figure S14c

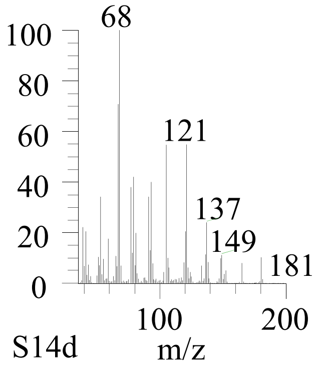

S14d

Fig. S15 Headspace GC-MS analysis of *N. benthamiana* infiltrated with *SdPAOMT* and *S. dorisiana* leaves. Mass spectra are shown of MePA, MeSA and MeBA peaks. a. m/z 180-181 MS, MePA analysis, b. m/z 152-153, MeSA analysis, c. m/z 136-137, MeBA analysis, d. GC-MS chromatogram of *S. dorisiana* leaf headspace. Methyl perillate is indicated by an asterisk. When specific m/z 92-93+120-121+152-153 are extracted from the *S. dorisiana* chromatogram, a small MeSA peak becomes visible at RT 13.06, NL 1.91E6 (in TIC the methylsalicylate peak is covered by a decanal peak at RT 12.95). However, the ratio MeSA: MePA in *S. dorisiana* is much smaller than in *N. benthamiana*, so probably in *S. dorisiana* substrate availability of SA is low.

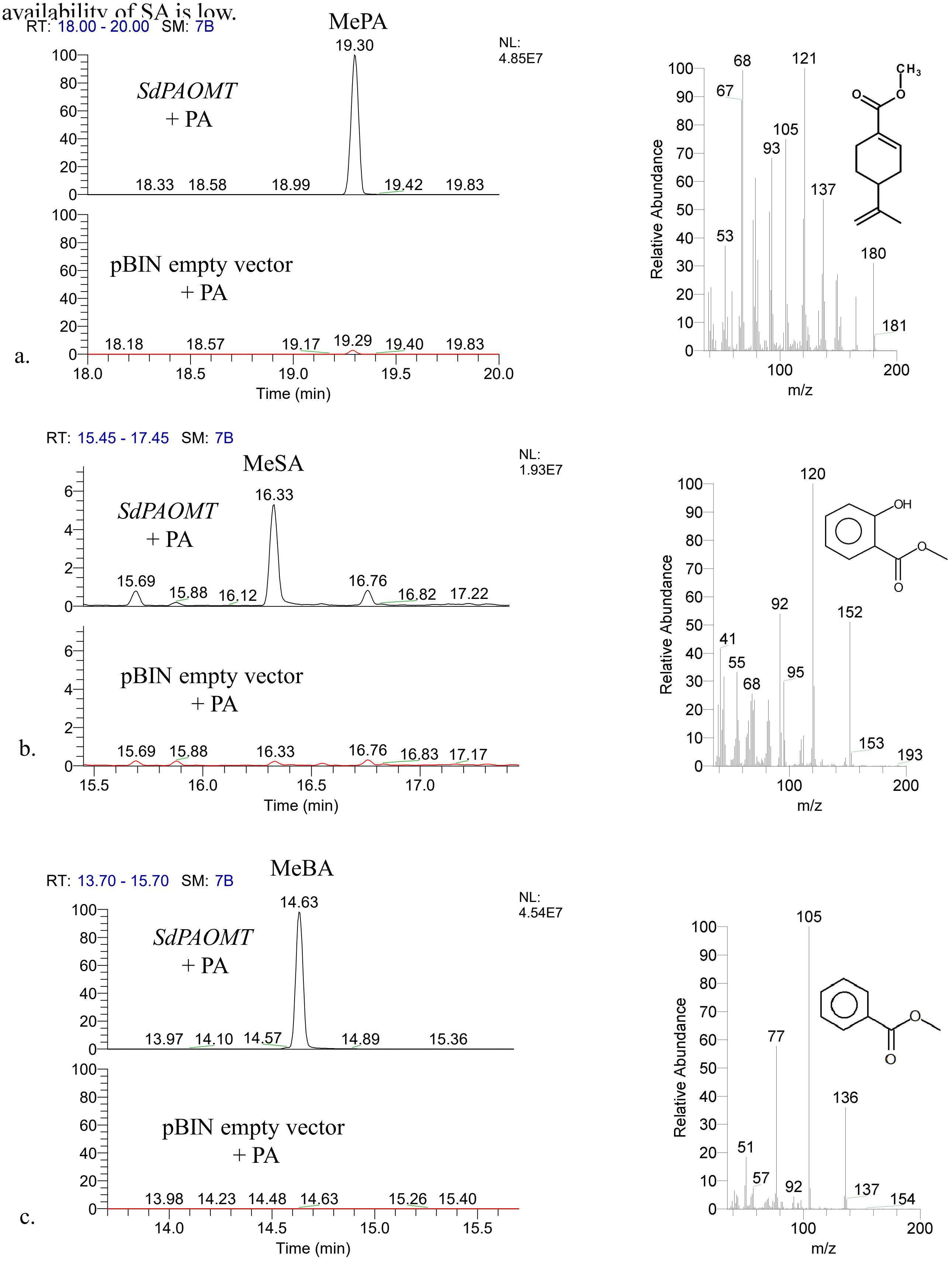

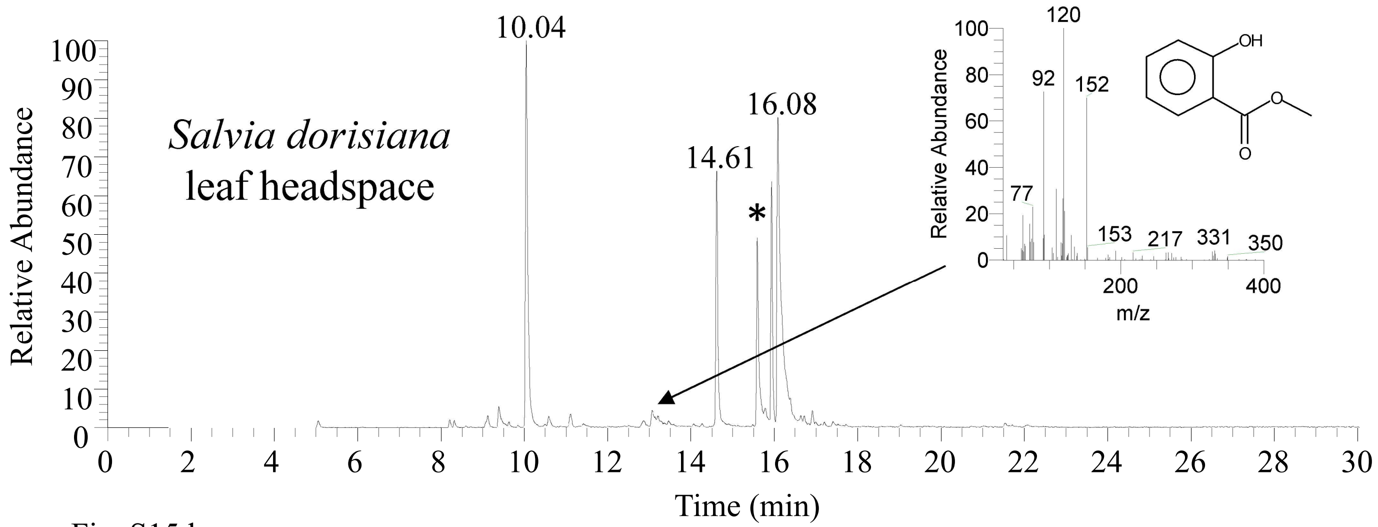

10.04
16.08
14.61
*
Salvia dorisiana
leaf headspace
Relative Abundance
Time (min)
120
92
152
77
153
217
331
350
m/z
OH
O
O

Fig. S15d.

Fig S16 Co-expression of *Picea abies* GPP synthase (*GPPS*) with *PfLS* in agroinfiltration of *N. benthamiana* increases limonene emission by 3-5 times

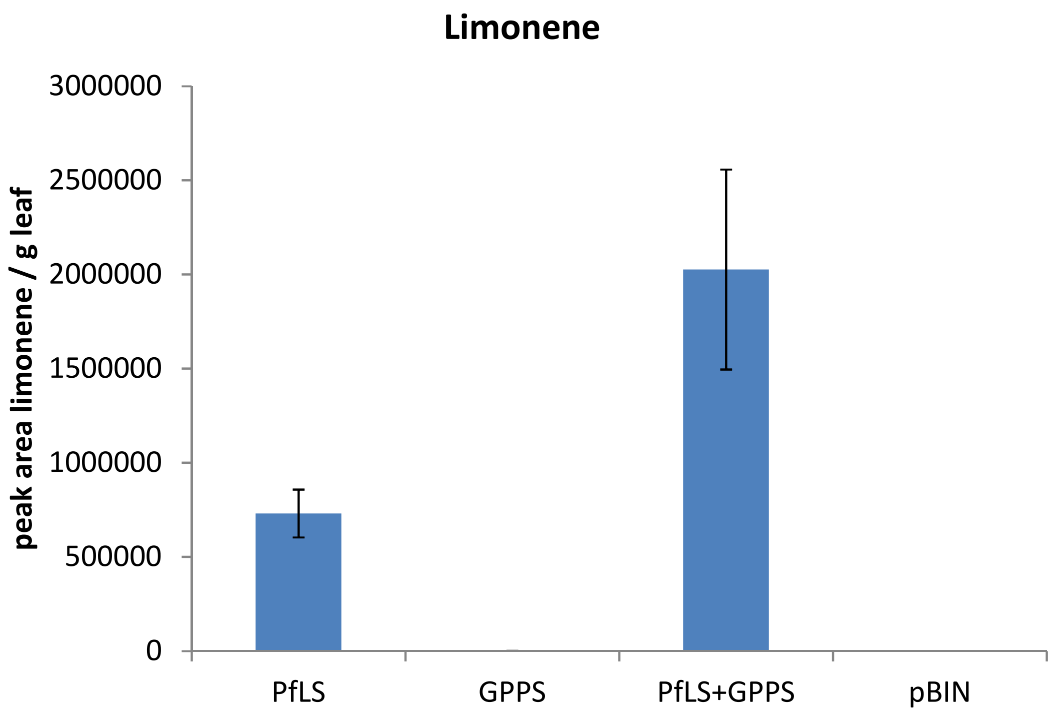

Figure S17. Agro-infiltration of methylperillate pathway genes (*SdLS, SdL7H, SdPOHDH and SdPAOMT*) results in production of perillyl aldehyde in the *N. benthamiana* leave headspace, which disappears when SdPOHDH is left out. a. GC-MS chromatograms of leave headspace m/z 149.5-150.5 NL 2.00E6, b. mass spectrum of perillyl aldehyde peak at RT 17.63, +c Full ms [35.00-400.00] c. mass spectrum of perillyl aldehyde reference compound

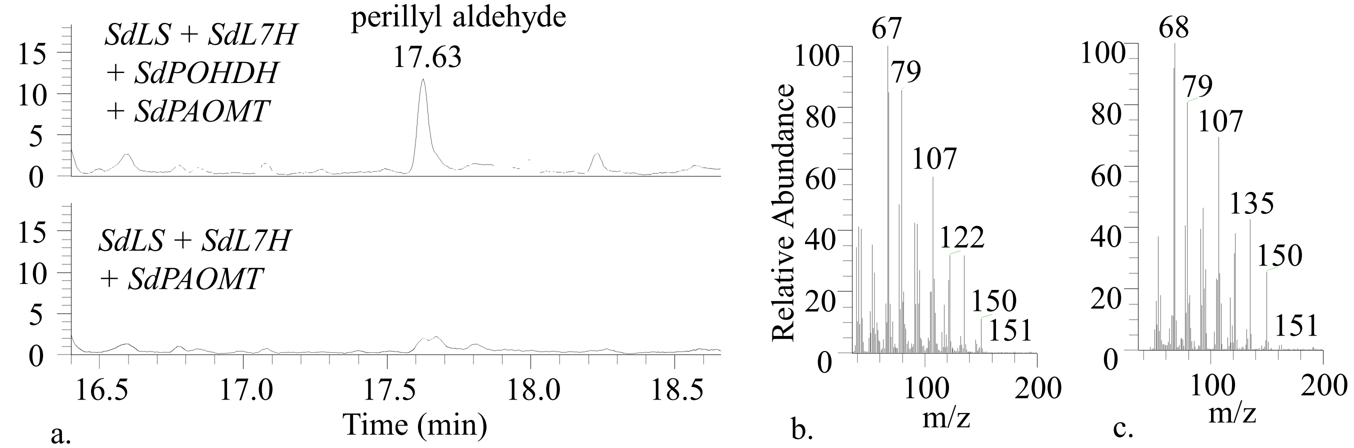

Figure S18 Methylperillate in agro-infiltrated *N. benthamiana* leaves headspace. GC-MS peak area methylperillate peak RT 19.26 m/z 180. Error bars indicate standard errors, N=3

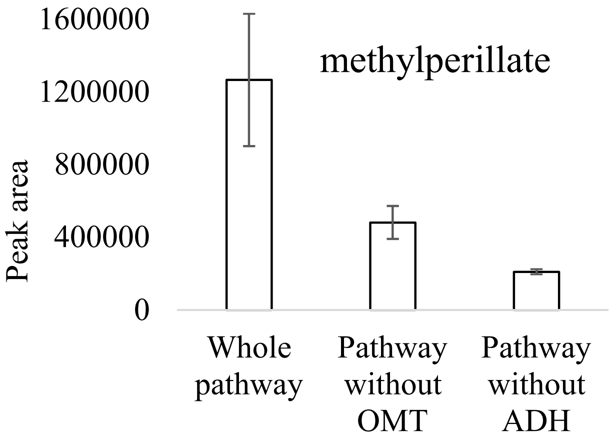

Figure S19: Reconstitution of the methylperillate biosynthesis pathway in *N. benthamiana*. Shown are LC-MS chromatograms of marker compounds representing plant metabolites of intermediates of the methylperillate pathway, and GC-MS chromatograms of methylperillate production in leaf headspace for different contruct combinations. Marker compounds perillyl alcohol (POH-Mk1 and Mk2), perillyl aldehyde (PAldH-Mk1), perillic acid (PA-Mk1, Mk2 and Mk3) or methylperillate (MPA). Chromatogram characteristics: vertical scales are the same for each marker compound: POH-Mk1: 100%=4.20E6 (m/z 355.1760-355.1770). POH-Mk2: 100%=1.22E6 (m/z 491.2130-491.2150). PAldH-Mk1 100%=6.86E4, (m/z 458.1950-458.1980). PA-Mk1: 100%=1.11E6 (m/z 373.1500-373.1530). PA-Mk2: 100%=4.67E5 (m/z 389.1450-389.1470). PA-Mk3: 100%=6.98E5 (m/z 535.2030-535.2050). Methylperillate: 100%=1.20E6, (m/z=180-181).

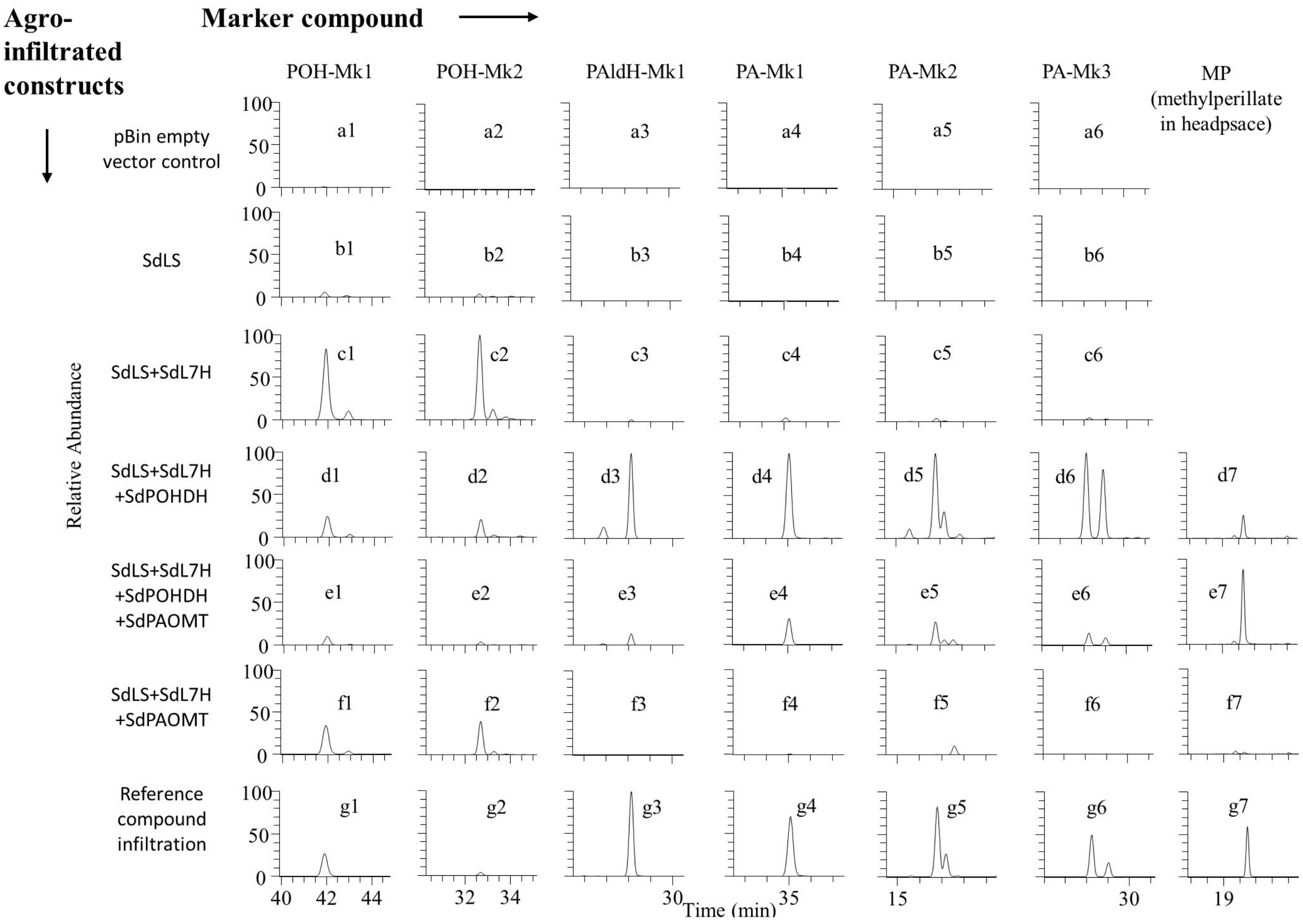

Supplement: eraa086_suppl_Supplementary_Figures_S1_S19 [file eraa086_suppl_supplementary_figures_s1_s19.pdf]
